# 3-Hydroxypropionic acid converts inflammatory macrophage glycolysis into mitochondrial oxidation through GAPDH carboxyethylation

**DOI:** 10.1016/j.isci.2026.116258

**Published:** 2026-06-04

**Authors:** Kefei Wu, Yankun Jia, Qi Lin, Fu Wang, Tianyue Liu, Mengjie Gao, Baodong Gao, Yumeng Zhu, Sifeng Xiong, Dong Sun, Ling Li, Huanyu Lu, Ping Zhu, Yue Zhai

**Affiliations:** 1 Department of Cell Biology of National Translational Science Center for Molecular Medicine, Fourth Military Medical University, Xi’an 710032, China; 2 State Key Laboratory of New Targets Discovery and Drug Development for Major Diseases, Xian 710032, China; 3 Department of Clinical Immunology, Xijing Hospital, Fourth Military Medical University, Xi’an 710032, China; 4 Xijing Innovation Research Institute, Fourth Military Medical University, Xi’an 710032, China; 5 Department of Occupational and Environmental Health and the Ministry of Education Key Lab of Hazard Assessment and Control in Special Operational Environment, School of Public Health, Fourth Military Medical University, Xi’an 710032, China

**Keywords:** molecular biology, immunology, cell biology

## Abstract

Macrophages dynamically reprogram their metabolic states in response to environmental stimuli, thereby exerting distinct immune functions. In our preliminary study, a gut microbiota-derived metabolite, 3-hydroxypropionic acid (3-HPA), is increased in chronic inflammation and generates cysteine carboxyethylated neoantigens. However, the role of such metabolite-induced modification in regulating the function of macrophages remains obscure. Here, we show that 3-HPA alleviates inflammation in a mouse model of sepsis and inhibits the macrophage inflammatory response. Mechanistically, 3-HPA induces glyceraldehyde-3-phosphate dehydrogenase (GAPDH) carboxyethylation, which promotes GAPDH degradation via the ubiquitin-proteasome pathway, thereby suppressing its enzymatic activity and expression to inhibit glycolysis. Concomitantly, reduced GAPDH activity elevates the NAD^+^/NADH ratio, which enhances mitochondrial oxidation by upregulating arginine biosynthesis and the TCA cycle pathway. Overall, our research reveals the mechanism by which GAPDH carboxyethylation mediates metabolic reprogramming and regulates inflammation during inflammatory macrophage activation.

## Highlights

• 3-HPA inhibits macrophage cytokine release
• 3-HPA induces GAPDH carboxyethylation and its ubiquitin-proteasome degradation
• GAPDH carboxyethylation inhibits GAPDH enzymatic activity
• 3-HPA switches macrophage glycolysis to mitochondrial oxidation via GAPDH carboxyethylation

## Graphical abstract

**Figure:**
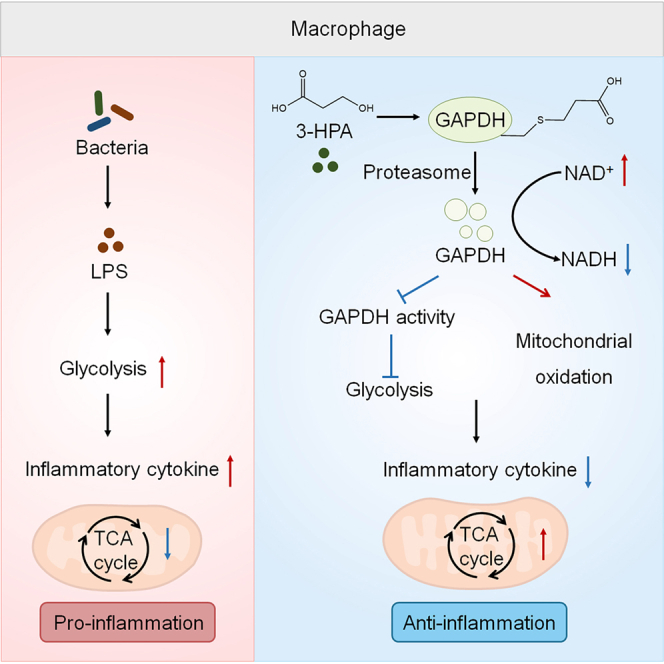

## Introduction

Macrophages play a critical role in the control of inflammatory status, and their functions can be easily affected by the cellular metabolic state.^1^^,^^2^ Pro-inflammatory macrophages induced by microbial products such as lipopolysaccharide (LPS) and other Toll-like receptor ligands utilize glycolysis to meet their energy demands and protein synthesis requirements. When the metabolic phenotype of macrophages shifts, for instance, toward oxidative phosphorylation or fatty acid oxidation, the subsequent release of inflammatory cytokines is affected.^3^ The metabolic adaptation of pro-inflammatory and anti-inflammatory macrophages can support macrophage activity and maintain their polarization under specific circumstances. However, the mechanisms underlying the metabolic shift from glycolysis to mitochondrial oxidation in macrophages and its functional consequences on their phenotype are incompletely understood.^4^^,^^5^

Glyceraldehyde-3-phosphate dehydrogenase (GAPDH) is a key enzyme in glycolysis, which catalyzes the conversion of glyceraldehyde-3-phosphate to 1,3-bisphosphoglyceric acid and mediates the formation of nicotinamide adenine dinucleotide (reduced form) (NADH) in the presence of nicotinamide adenine dinucleotide (NAD^+^) and inorganic phosphate.^6^ The significant upwelling of glycolysis and the increase in GAPDH activity are characteristics of activated macrophages, which make GAPDH an attractive anti-inflammatory target in macrophages.

An increasing number of studies have shown that the function of GAPDH is regulated by post-translational modifications (PTMs). Endogenous metabolites, as substrates for PTMs of proteins, are emerging as a key means of regulating immunity.^7^ For example, dimethyl fumarate covalently modifies cysteine residues of GAPDH, which down-regulates aerobic glycolysis in activated macrophages and mediates anti-inflammatory effects.^8^ 4-octyl itaconate alkylates cysteine on GAPDH and decreases its enzyme activity.^9^ GAPDH undergoes malonylation, leading to its dissociation from *TNF-α* mRNA to promote inflammation.^10^

Our previous work has proved that cysteine carboxyethylation could be induced by gut microbiota-derived metabolites 3-hydroxypropionic acid (3-HPA)^11^^,^^12^^,^^13^ and generate post-translational modified neoantigens.^14^ Meanwhile, we found that 3-HPA has the potential for broader modification targets, and it may also have important, yet unknown, functions in regulating immune cells. In this study, we show that 3-HPA induces GAPDH carboxyethylation, which promotes GAPDH degradation via the ubiquitin-proteasome pathway, thereby suppressing its enzymatic activity and expression to inhibit glycolysis. Reduced GAPDH activity elevates the NAD^+^/NADH ratio, which enhances mitochondrial oxidation by upregulating arginine biosynthesis and the TCA cycle pathway. Through the carboxyethylation modification of GAPDH, the metabolism of macrophages is transformed from glycolysis to mitochondrial oxidation, thereby exerting anti-inflammatory effects and alleviating sepsis.

## Results

### 3-HPA alleviated sepsis and inhibited the release of inflammatory cytokines from macrophages

Although 3-HPA was reported to induce HLA-restricted autoimmunity by modifying cysteine carboxyethylation to form neoantigens,^14^ it was still not clear whether 3-HPA affected innate immunity by influencing the function of macrophages. Considering macrophages play a significant regulatory role in the immune dysfunction of sepsis,^15^ we used cecal ligation and perforation (CLP) mouse models and LPS-induced bacteremia models to evaluate the effect of 3-HPA on innate immunity (Figure S1A; Figure S2A). Survival analysis showed that 3-HPA treatment significantly improved the survival probability of CLP mice (Figure S1B). In line with this, 3-HPA reduced the serum level of IL-6 (Figure S1C) and spleen weight (Figure S1D) in CLP mice, indicating alleviated systemic inflammation and organ damage. The liver and lung tissues' histopathological examination (organs that are often damaged in sepsis) revealed that in the CLP group, the hepatic sinusoids of the liver were dilated, with obvious hemorrhage. However, 3-HPA treatment alleviated liver injury. Additionally, in the CLP group, the alveolar structure of the lungs was damaged, a large number of inflammatory cells infiltrated, and exudation occurred in the alveolar cavities. 3-HPA alleviated lung damage (Figure S1E). In the LPS model, RT-PCR results showed that 3-HPA treatment significantly reduced the level of *Tnf-α* and *Il-6* mRNA in the liver (Figure S2B), while there was no significant change in *Il-1β*. In the LPS group, H&E showed that inflammatory cell infiltration and tissue edema occurred in the liver. 3-HPA treatment alleviated LPS-induced liver injury. The alveolar structure of the lungs in the LPS group was damaged, with a large number of inflammatory cells infiltrating and exudation in the alveolar cavities. Compared with this, 3-HPA treatment alleviated lung and liver damage (Figure S2C). These results suggested that 3-HPA alleviated the sepsis-induced inflammatory damage.

**Figure 1:**
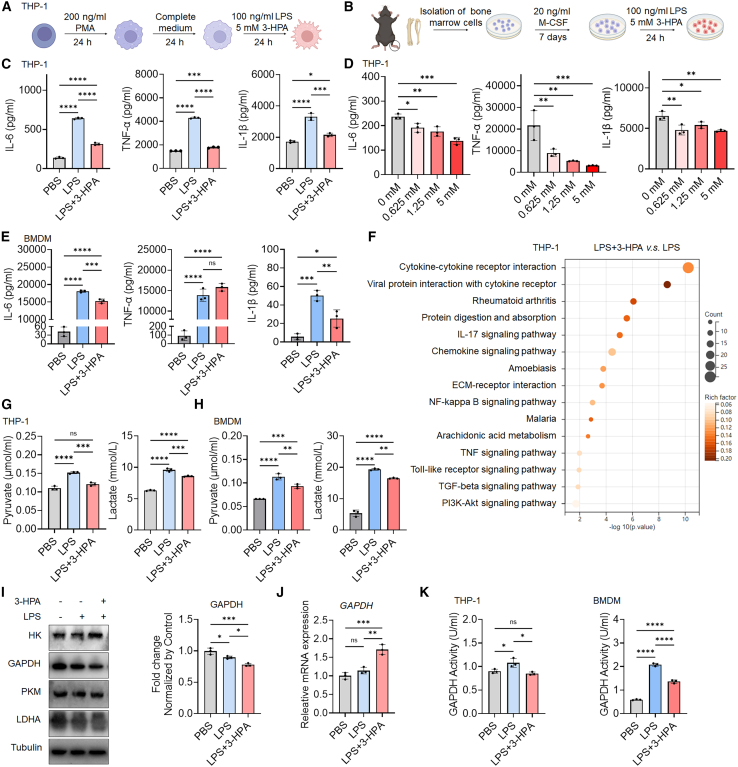
3-HPA inhibits the secretion of inflammatory factors and glycolysis in macrophages (A and B) Schematic diagram of THP-1 cell (A) and BMDMs (B) activation into pro-inflammatory macrophages. Created with BioRender.com. (C) The concentrations of IL-6, TNF-α, and IL-1β in THP-1 cell supernatants were quantified by ELISA after 24 h treatment with LPS (100 ng/mL), or LPS+3-HPA (5 mM). Data are the means ± SD and *n* = 3 per group. Statistical significance was determined using one-way ANOVA followed by Tukey’s multiple comparisons test with *∗p* < 0.05, *∗∗p* < 0.01, *∗∗∗p* < 0.001, and *∗∗∗∗p* < 0.0001. (D) The concentrations of IL-6, TNF-α, and IL-1β in supernatants of THP-1 cells treated with different concentrations of 3-HPA (0, 0.625, 1.25, 5 mM) followed by LPS stimulation. Data are the means ± SD and *n* = 3 per group. Statistical significance was determined using one-way ANOVA followed by Dunnett’s multiple comparisons test *∗p* < 0.05, *∗∗p* < 0.01, and *∗∗∗p* < 0.001. (E) The concentrations of IL-6, TNF-α, and IL-1β in BMDM cell supernatants were quantified by ELISA after 24 h treatment with LPS (100 ng/mL) or LPS+3-HPA (5 mM). Data are the means ± SD and *n* = 3 per group. Statistical significance was determined using one-way ANOVA followed by Tukey’s multiple comparisons test with *∗p* < 0.05, *∗∗p* < 0.01, *∗∗∗p* < 0.001, and *∗∗∗∗p* < 0.0001. (F) Bubble plot of KEGG pathway enrichment analysis for differentially expressed genes between LPS (100 ng/mL) and LPS+3-HPA (5 mM) treated in THP-1 cells. The size of each bubble represents the number of differentially expressed genes, and the color indicates the enrichment factor. (G and H) Pyruvate and lactate levels in THP-1 cells (G) and BMDMs (H) treated with LPS (100 ng/mL), or LPS+3-HPA (5 mM). (I) Immunoblots of protein expression levels of HK, GAPDH, PKM, and LDHA in THP-1 cells treated with LPS (100 ng/mL), or LPS+3-HPA (5 mM), and quantitative results of GAPDH. (J) The mRNA levels of *GAPDH* in THP-1 cells treated with LPS (100 ng/mL) or LPS+3-HPA (5 mM). (K) GAPDH activity assay in THP-1 cells and BMDMs treated with PBS, LPS (100 ng/mL), or LPS+3-HPA (5 mM). Data in (G–K) are the means ± SD and *n* = 3 per group. Statistical significance was determined using one-way ANOVA followed by Tukey’s multiple comparisons test with *∗p* < 0.05, *∗∗p* < 0.01, *∗∗∗p* < 0.001, and *∗∗∗∗p* < 0.0001; ns, not significant.

To further explore the cellular mechanism, we focused on macrophages, the central mediators of inflammatory cytokine production in sepsis. We stimulated THP-1 (Figure 1A) and mouse bone marrow-derived macrophages (BMDMs) (Figure 1B) with LPS to induce pro-inflammatory macrophages, treated with or without 3-HPA for 24 h, and then detected the protein expressions of inflammatory factors. ELISA assays demonstrated that 3-HPA markedly decreased the secretion of IL-6, TNF-α, and IL-1β in THP-1 cells (Figure 1C), and the levels of secreted inflammatory factors gradually decreased with the increase of 3-HPA level in a concentration-dependent manner (Figure 1D). In BMDMs, the protein levels of IL-6 and IL-1β decreased (Figure 1E). In addition, 3-HPA promoted the transcription of anti-inflammatory marker genes (such as *IL-10*, *TGFB1*, and *ARG1*) in THP-1 and BMDMs (Figures S3A and S3B). To exclude the possibility that the anti-inflammatory effects of 3-HPA were due to cytotoxicity, we assessed cell viability using the CCK-8 assay. The results showed that 3-HPA treatment at concentrations up to 5 mM did not affect the viability of THP-1 cells or BMDMs under either basal or LPS-stimulated conditions (Figures S3C and S3D). We conducted RNA sequencing (RNA-seq) to investigate the effect of 3-HPA on macrophage function under LPS stimulation. KEGG pathway enrichment analysis indicated that under 3-HPA treatment, not only were all differentially expressed genes (DEGs) significantly enriched in the cytokine-cytokine receptor interaction pathway (Figure 1F), but this pathway was also prominently enriched among the downregulated gene set (Figure S3E). Collectively, the findings that 3-HPA ameliorates pathological responses in septic mice and significantly reduces the secretion of inflammatory cytokines from macrophages suggest that 3-HPA exerts an inhibitory effect on the release of inflammatory cytokines by macrophages.

Since the metabolic characteristics of macrophages are closely related to the inflammatory phenotype,^16^ we further investigated the metabolic mechanism underlying the anti-inflammatory effect of 3-HPA. In the glycolytic pathway, 3-HPA decreased the content of pyruvate and lactate in both THP-1 and BMDM cells under LPS stimulation (Figures 1G and 1H).

The efficiency of a metabolic pathway is determined by the expression and activity of its key rate-limiting metabolic enzymes. Previous studies have shown that hexokinase (HK), pyruvate kinase muscle (PKM), and GAPDH, which are the key rate-limiting metabolic enzymes in the glycolytic pathway, play a crucial role in macrophage metabolic reprogramming.^17^ Subsequently, the protein expression of enzymes in the glycolytic pathway from the pro-inflammatory macrophages was examined. Notably, immunoblot detection showed that 3-HPA treatment significantly decreased the protein level of GAPDH (Figure 1I). However, at the mRNA level, 3-HPA increased *GAPDH* mRNA transcription (Figure 1J). RNA-sequencing results showed that 3-HPA treatment had no effect on the mRNA levels of *HK1*, *PFKM*, *PKM*, and *LDHA* (Figure S4A). In addition, 3-HPA also significantly reduced GAPDH activity in THP-1 and BMDMs (Figure 1K). Therefore, 3-HPA-mediated impaired glycolytic function in macrophages may be related to the decreased expression and activity of GAPDH.

### GAPDH was modified with carboxyethylation at cys 247

**Figure 2:**
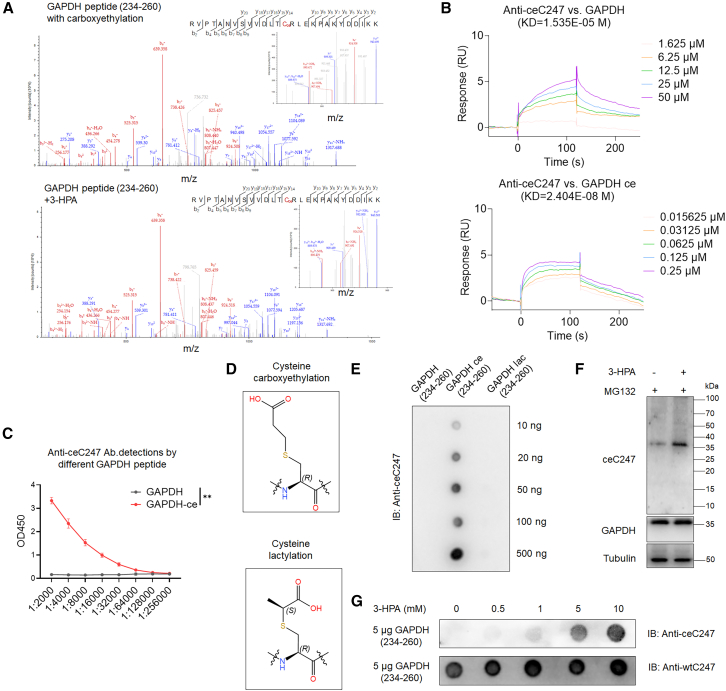
GAPDH is modified with carboxyethylation at Cys 247 (A) Mass spectrometry analysis of GAPDH peptide (234–260) with carboxyethylation and 3-HPA (5 mM) incubated with the GAPDH peptide (234–260) at 37 °C for 4 h. (B) SPR analysis of the affinity of the anti-ceC247 antibody for the carboxyethylation modified GAPDH peptide (234–260) and unmodified GAPDH peptide (234–260). (C) ELISA-based binding curve of anti-ceC247 antibody to modified GAPDH peptide (GAPDH ce (234–260)) and unmodified GAPDH peptide (234–260). Data are the means ± SD and *n* = 3 per group. Statistical significance was determined using two-way ANOVA followed by *∗∗p* < 0.01. (D) Chemical structures of cysteine carboxyethylation and cysteine lactylation. (E) Unmodified GAPDH peptide (234–260), carboxyethylated peptide (GAPDH ce (234–260)), and lactylated peptide (GAPDH lac (234–260)) were tested with the anti-ceC247 antibody in dot blot assays. (F) Immunoblots of lysates from 293 T cells overexpressing GAPDH, which were treated with 5 mM 3-HPA and 5 μM MG132. The blots were probed with the anti-ceC247 antibody. (G) 3-HPA incubated with the GAPDH peptide (234–260) at 37 °C for 4 h. An anti-ceC247 antibody and the anti-wtC247 antibody were used in dot blot assays.

3-HPA is an endogenous metabolite mainly derived from microorganisms and food, which can be modified by cysteine carboxyethylation. Our previous reported data showed that GAPDH Cys247 was modified with carboxyethylation in PBMCs from patients with ankylosing spondylitis (AS). Then, to test 3-HPA-mediated GAPDH Cys247 carboxyethylation, we co-incubated 3-HPA with the GAPDH peptide segment at 37 °C for 4 h. Liquid chromatograph mass spectrometer (LC-MS/MS) spectra showed that the GAPDH peptide containing cysteine 247 was covalently modified by 3-HPA (+72.021Da) (Figure 2A).

To confirm the occurrence of cysteine carboxyethylation, a specific antibody against carboxyethylated GAPDH Cys247 (GAPDH-ce) was prepared in BALB/C mice. The surface plasmon resonance (SPR) detection indicated that GAPDH-ce247 antibody (anti-ceC247) had a high affinity and specificity for GAPDH-ce (KD = 2.404 E^−08^ M) (Figure 2B). ELISA experiments also demonstrated that anti-ceC247 antibody had a high affinity and specificity for GAPDH-ce (Figure 2C). Although cysteine lactylation and cysteine carboxythylation had the same mass bias of +72.021,^18^ the structures of lactation modification and carboxyethylation modification are different (Figure 2D). We also examined the affinity of the anti-ceC247 antibody for the lactylated peptide of GAPDH Cys247 (GAPDH-lac), and dot-blot results showed that the anti-ceC247 antibody did not recognize GAPDH-lac (Figure 2E). To investigate GAPDH carboxyethylation, we first utilized 293 T cells, as they are widely employed in PTM research due to their high transfection efficiency and robust protein expression. When GAPDH was transiently expressed in 293 T cells in the presence of 3-HPA, the carboxyethylated GAPDH was clearly detected by immunoblot using the anti-ceC247 antibody (Figure 2F). We incubated the unmodified GAPDH peptide with different concentrations of 3-HPA at 37 °C for 4 h, and detected them with anti-ceC247 antibody and unmodified antibody. The result showed that with the increase of 3-HPA concentration, the formation of carboxyethylated GAPDH gradually increased (Figure 2G). These results indicated that GAPDH carboxyethylation modification exists within the cells, and 3-HPA treatment induced GAPDH Cys247 carboxyethylation.

To investigate whether 3-HPA induces GAPDH carboxyethylation *in vivo*, we utilized an LPS-induced mouse model of sepsis. Peritoneal macrophages were isolated from mice in the LPS group and the LPS+3-HPA group, and treated with the proteasome inhibitor to preserve the modified protein. GAPDH carboxyethylation at Cys247 was then assessed using anti-ceC247 antibody. Immunoblot analysis revealed that GAPDH carboxyethylation levels were significantly increased in peritoneal macrophages from the LPS-induced model treated with 3-HPA compared to those from mice treated with LPS alone (Figure S2D). These results demonstrate that 3-HPA promotes GAPDH Cys247 carboxyethylation in peritoneal macrophages under inflammatory conditions *in vivo*.

### The carboxyethylation of GAPDH increased the ubiquitination level of GAPDH K48 and degraded it through the proteasome pathway

**Figure 3:**
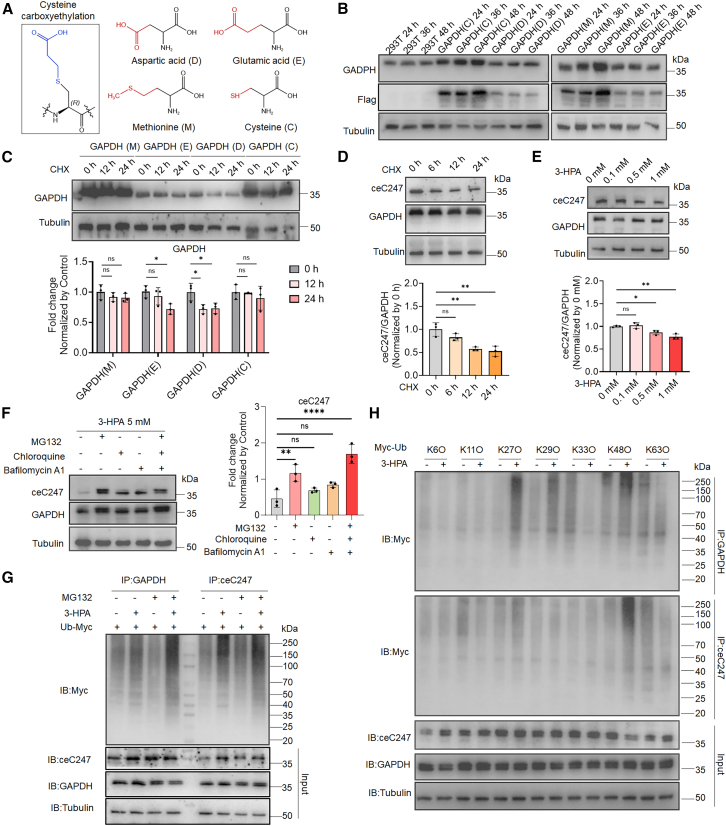
3-HPA-induced carboxyethylation of GAPDH promotes its degradation through the ubiquitin-proteasome pathway (A) Chemical structure of carboxyethylated cysteine (left) and structures of aspartic acid (D), glutamic acid (E), methionine (M), and cysteine (C). (B) Immunoblot of GAPDH after transient transfection of flag-tagged GAPDH(C), GAPDH(D), GAPDH(M), GAPDH(E) plasmid in 293 T cells at 24 h, 36 h, and 48 h. (C) Immunoblot of GAPDH after CHX treatment. The GAPDH antibody was used to compare the degradation rates of GAPDH(M), GAPDH(E), GAPDH(D), and GAPDH(C). Data are the means ± SD and *n* = 3 per group. Statistical significance was determined using one-way ANOVA followed by Dunnett’s multiple comparisons test *∗p* < 0.05; ns, not significant. (D) Immunoblot and quantitative analysis of GAPDH ce after CHX treatment. The anti-ceC247 antibody and anti-GAPDH antibody were used to compare the degradation rates of carboxyethylated GAPDH. Data are the means ± SD and *n* = 3 per group. Statistical significance was determined using one-way ANOVA followed by Dunnett’s multiple comparisons test *∗∗p* < 0.01; ns, not significant. (E) Immunoblot and quantitative analysis of GAPDH ce after 3-HPA treatment. The anti-ceC247 antibody and anti-GAPDH antibody were used to compare the content of carboxyethylated GAPDH and total GAPDH. Data are the means ± SD and *n* = 3 per group. Statistical significance was determined using one-way ANOVA followed by Dunnett’s multiple comparisons test *∗p* < 0.05; *∗∗p* < 0.01; ns, not significant. (F) Immunoblot and quantitative analysis of GAPDH ce in 293 T cells treated with 3-HPA (5 mM) combined with proteasomal inhibitor MG132, autophagic inhibitor Chloroquine, or lysosomal inhibitor Bafilomycin A1. Data are the means ± SD and *n* = 3 per group. Statistical significance was determined using one-way ANOVA followed by Dunnett’s multiple comparisons test *∗∗p* < 0.01; *∗∗∗∗p* < 0.0001; ns, not significant. (G) Immunoprecipitation of GAPDH or GAPDH ce followed by immunoblotting for Myc in 293 T cells transfected with Myc-Ub. Cells were treated with 3-HPA (5 mM) and MG132 (5 μM) for 24 h. (H) Immunoprecipitation of GAPDH or GAPDH ce followed by immunoblotting for Myc in 293 T cells transfected with Myc-Ub mutants (K6O, K11O, K27O, K29O, K33O, K48O, K63O). Cells were treated with 3-HPA (5 mM) for 24 h.

To further investigate the function of GAPDH cys247 carboxyethylation, we constructed mutant plasmids that simulated the modified structure. Cysteine was mutated to aspartic acid (GAPDH-D) and glutamic acid (GAPDH-E), respectively, to simulate the carboxyl group structure modified by carboxyethylation. Cysteine was mutated to methionine (GAPDH-M) to simulate the carboxyethylated modified thiosulfide bond (Figure 3A). It is worth noting that when the plasmids were transiently transfected into 293 T cells, the GAPDH protein expression levels of the cells transfected with GAPDH(D) and GAPDH(E) plasmids were lower than those transfected with GAPDH(M) and GAPDH(C) plasmids (Figure 3B). Subsequently, we constructed a 293 T cell line with stable plasmid transfection. The expression level of GAPDH protein in the cells transfected with the GAPDH(D) plasmid was significantly lower than that in the cells transfected with GAPDH(M) and those without mutation, but there was no difference in the *GAPDH* mRNA level (Figures S5A and S5B).

We next examined the stability of GAPDH and GAPDH-ce. Compared to GAPDH(C), GAPDH(D) and GAPDH(E) were more easily degraded in cycloheximide (CHX)-treated cells (Figure 3C). Additionally, compared to unmodified GAPDH, GAPDH-ce was more easily degraded in CHX-treated cells (Figure 3D). The anti-ceC247 antibody was used to detect the effect of 3-HPA treatment concentration on the expression of GAPDH-ce. The results showed that the expression of GAPDH-ce decreased gradually with the increase of 3-HPA concentration (Figure 3E). These results suggested that the carboxythylation modification of GAPDH may lead to GAPDH protein degradation. In addition, adding the proteasome inhibitor MG132 to the cells efficiently stabilized the protein level of both GAPDH-ce and GAPDH. However, the reduced level of GAPDH-ce cannot be reversed by the treatment of serine and thiol protease inhibitor leupeptin, lysosomal inhibitor bafilomycin A1 (Baf.A), and autophagy lysosomal inhibitor chloroquine (Figure 3F; Figure S5C).

Subsequently, we transfected the ubiquitin plasmid with Myc-tag into 293 T cells and found that 3-HPA treatment significantly enhanced the ubiquitination of GAPDH and GAPDH-ce (Figure 3G). Screening of ubiquitin mutants of the potential lysine ubiquitylation type showed that 3-HPA increased K48O (Ub with an intact Lys48 residue only) ligation to GAPDH and GAPDH-ce (Figure 3H). Meanwhile, we used anti-ubiquitin (linkage-specific K48) antibody to detect the co-immunoprecipitation of protein complexes with anti-ceC247 antibody and unmodified GAPDH antibody, and the results showed that 3-HPA enhanced the K48 ubiquitination of GAPDH and GAPDH-ce (Figure S5D). These results showed that the carboxyethylation of GAPDH increased the ubiquitination level of GAPDH K48, thereby making GAPDH more readily degraded through the proteasome pathway.

To explore the structural basis of GAPDH function and its modulation by 3-HPA, we performed *in silico* analysis. Prediction tools (mCSM, SDM, DUET) indicated that the C to E mutation at position 247 of GAPDH may lead to structural instability (Figure S6A). Additionally, 3D structural modeling and molecular dynamics simulations showed that the structural differences between GAPDH(E) and GAPDH(C) are due to mutations altering the conformation of the 183–207 loop region (Figures S6B and S6C). In addition, molecular dynamics simulations show that the GAPDH(C) structure converges significantly faster. Compared with the GAPDH(E), the GAPDH(C) structure was more stable. The GAPDH(C) structure had milder local residue fluctuations, and the GAPDH(E) mutant had slightly higher local flexibility (Figures S6D and S6E). These results showed that the carboxyethylation modification of GAPDH affected GAPDH's structural stability.

In addition, immunofluorescence detected that there were differences in the localization of GAPDH and GAPDH-ce in THP-1 cells. The GAPDH molecule was mainly located in the nucleus and cytoplasm, while the GAPDH-ce molecule was mainly located in the cytoplasm (Figure S7A). The nuclear and cytoplasmic separation of THP-1 cells also further confirmed that GAPDH was present in the nucleus and cytoplasm, while GAPDH-ce was mainly located in the cytoplasm (Figure S7B).

### 3-HPA increased the ratio of NAD^+^/NADH and enhanced mitochondrial oxidation

**Figure 4:**
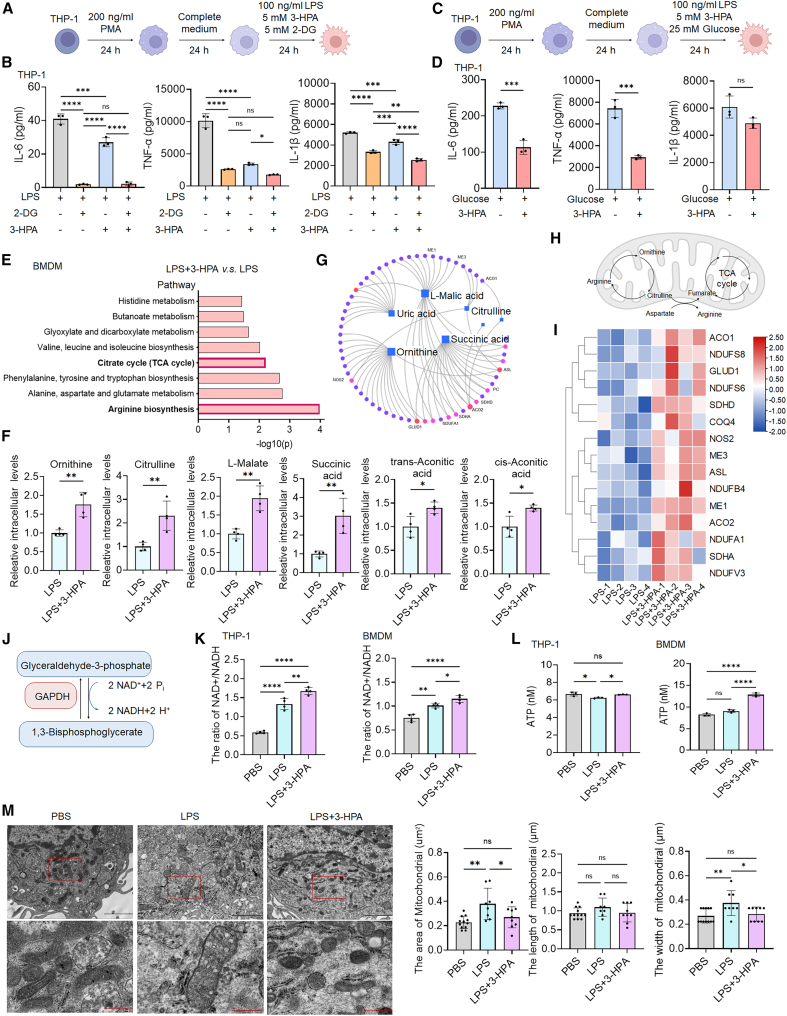
3-HPA enhances the metabolite content of the TCA cycle and mitochondrial oxidation (A) Schematic diagram of THP-1 with 2-DG treatment. Created with BioRender.com. (B) Concentrations of IL-6, TNF-α, and IL-1β in supernatants of THP-1 cells treated with LPS, LPS+3-HPA, LPS+2-DG, or LPS+3-HPA+2-DG. Data are the means ± SD and *n* = 3 per group. Statistical significance was determined using one-way ANOVA followed by Tukey’s multiple comparisons test with *∗p* < 0.05, *∗∗p* < 0.01, *∗∗∗p* < 0.001, and *∗∗∗∗p* < 0.0001; ns, not significant. (C) Schematic diagram of THP-1 with high glucose treatment. Created with BioRender.com. (D) Concentrations of IL-6, TNF-α, and IL-1β in supernatants of THP-1 cells treated with LPS+3-HPA, LPS+3-HPA+glucose. Data are the means ± SD and *n* = 3 per group. Statistical significance was determined using unpaired Student’s *t* test with *∗∗∗p* < 0.001; ns, not significant. (E) KEGG pathway enrichment analysis of metabolic pathways in BMDM cells treated with LPS or LPS+3-HPA. (F) Relative abundance of metabolite (ornithine, citrulline, L-malate, succinic acid, trans-aconitic acid, cis-aconitic acid) in BMDM cells treated with LPS or LPS+3-HPA. Data are the means ± SD and *n* = 4 per group. Statistical significance was determined using unpaired Student’s *t* test with *∗p* < 0.05; *∗∗p* < 0.01. (G) Correlation network of metabolites and genes in the metabolic pathway. Nodes represent metabolites (blue squares) and genes (colored circles). Gray edges indicate pairwise correlations between metabolites and genes. The colors similarly represent expression levels, with red typically indicating higher expression and blue indicating lower expression compared to the mean. (H) Schematic diagram of arginine metabolism and the TCA cycle. (I) Heatmap of mitochondrial oxidation-related gene expression associated with differentially expressed metabolites. Red indicates relatively high gene expression, while blue indicates relatively low gene expression within each row. (J) Schematic diagram of the catalytic function of the GAPDH enzyme. (K) Concentrations of the NAD^+^/NADH ratio in THP-1 cells and BMDM cells treated with PBS, LPS, or LPS+3-HPA. Data are the means ± SD and *n* = 4 per group. Statistical significance was determined using one-way ANOVA followed by Tukey’s multiple comparisons test with ∗*p* < 0.05, ∗∗*p* < 0.01, and ∗∗∗∗*p* < 0.0001; (L) ATP concentrations in THP-1 cells and BMDMs treated with PBS, LPS, or LPS+3-HPA. Data are the means ± SD and *n* = 3 per group. Statistical significance was determined using one-way ANOVA followed by Tukey’s multiple comparisons test with ∗*p* < 0.05; ∗∗∗∗*p* < 0.0001; ns, not significant. (M) Representative images and mitochondrial analysis of BMDM cells treated with PBS, LPS, or LPS+3-HPA. Scale bars, 1 μm (upper) and 0.25 μm (lower). For mitochondrial number, *n* = 8–12 (*n* = 12 for PBS, *n* = 8 for LPS, *n* = 9 for LPS+3-HPA group).

Based on 3-HPA-mediated GAPDH degradation, to determine whether the metabolite 3-HPA affected macrophage inflammatory cytokine secretion via the glycolytic pathway, we manipulated glycolytic flux by supplementing with high glucose and inhibiting glycolysis using 2-DG. In THP-1 cells, 2-DG inhibition of glycolysis reduces the production of cytokines. Both 3-HPA and 2-DG can inhibit the secretion of inflammatory factors (IL-6, TNF-α, IL-1β). When 3-HPA and 2-DG are added simultaneously, compared with adding 2-DG alone, 3-HPA can still further inhibit the secretion of IL-1β (Figures 4A and 4B). In addition, after supplementing with high glucose, 3-HPA significantly reduced the secretion of inflammatory factors (IL-6, TNF-α) (Figures 4C and 4D). These findings suggest that 3-HPA exerted its anti-inflammatory effect through the glycolytic pathway, but its effect is not confined to this single metabolic axis.

To describe 3-HPA-mediated systemic metabolic pathways, we performed BMDM cell-targeted metabolomics assays. The results showed that the differentially up-regulated metabolites in 3-HPA treatment were mainly enriched in arginine biosynthesis and the TCA cycle pathway (Figure 4E). 3-HPA treatment significantly upregulated the contents of ornithine, citrulline, L-malate, succinic acid, trans-Aconitic acid, and cis-Aconitic acid (Figure 4F). Furthermore, the genes related to the upregulation of metabolites also increased significantly (Figures 4G–4I).

GAPDH is a key enzyme that catalyzes the conversion of glyceraldehyde-3-phosphate to 1,3-bisphosphoglyceric acid and mediates the formation of NADH in the presence of NAD^+^ and inorganic phosphate (Figure 4J).^6^ Since the carboxyethylation of GAPDH mediated by 3-HPA decreased the content and activity of GAPDH, we subsequently examined the function of GAPDH and detected the contents of NAD^+^ and NADH. In LPS-induced THP-1 and BMDM pro-inflammatory macrophages, 3-HPA treatment increased the NAD^+^/NADH ratio (Figure 4K). The ratio of NAD^+^ to NADH is crucial for many metabolic reactions, including central carbon metabolism, lipid metabolism, and amino acid metabolism.^19^ Studies have proved that NAD^+^/NADH drives the transition of acute inflammation from early glycolysis to later fatty acid oxidation by activating SIRT1 and SIRT6 in TLR4-stimulated THP-1 cells. Among them, SIRT1 activation is NAD^+^/NADH dependent and increases the expression of mitochondrial and lipid metabolism genes by activating the PGC-1 pathway, thereby modulating mitochondrial function and lipid metabolism.^20^^,^^21^Then, we found that in LPS-induced THP-1 and BMDM, 3-HPA promoted the transcription of CPT1 (Figures S8A and S8B). In addition, 3-HPA significantly increased the ATP levels of LPS-stimulated THP1 and BMDMs (Figure 4L). To assess the effect of 3-HPA on glycolysis and oxidative phosphorylation in BMDMs, we performed Seahorse analyses under LPS-stimulated conditions. Mitochondrial stress test revealed that 3-HPA treatment increased basal respiration, proton leak, and ATP production (Figure S8C). In contrast, the glycolytic stress test demonstrated that 3-HPA significantly reduced glycolysis, glycolytic capacity, and glycolytic reserve (Figure S8D). Furthermore, tetramethylrhodamine methyl ester (TMRM) staining followed by flow cytometry analysis showed that 3-HPA increased the mitochondrial membrane potential in both THP-1 cells and BMDMs (Figure S8E). These results indicate that 3-HPA promotes a metabolic shift from glycolysis toward oxidative phosphorylation in activated macrophages. And 3-HPA attenuated LPS-induced mitochondrial swelling in macrophages, as demonstrated by electron microscopy (Figure 4M). These results suggested that 3-HPA increased mitochondrial oxidation in macrophages.

Altogether, we concluded that 3-HPA inhibited GAPDH activity, thereby increasing the NAD^+^/NADH ratio and enhancing mitochondrial oxidation.

### 3-HPA inhibited inflammatory macrophage phenotype and metabolic function by GAPDH cys247 carboxyethylation

**Figure 5:**
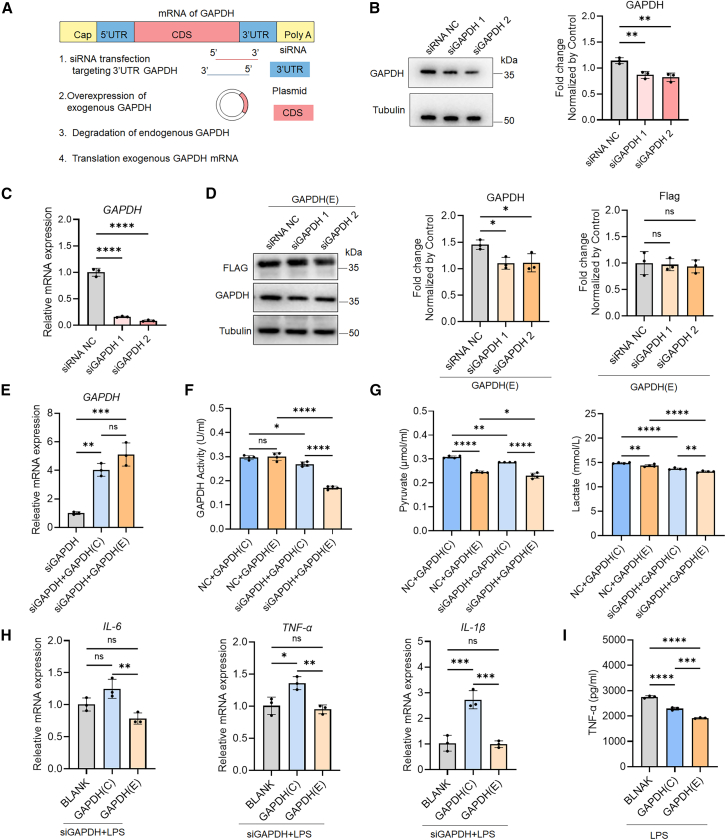
GAPDH carboxyethylation inhibited macrophage glycolysis and the release of inflammatory factors (A) Schematic workflow illustrating the strategy of silencing endogenous GAPDH via 3′UTR-targeting siRNA and overexpressing exogenous GAPDH. (B) Immunoblot and quantitative analysis of GAPDH protein in 293 T cells transfected with GAPDH 3′UTR-targeting siRNAs (siGAPDH 1, siGAPDH 2) or siRNA NC. Data are the means ± SD and *n* = 3 per group. Statistical significance was determined using one-way ANOVA followed by Dunnett’s multiple comparisons test *∗∗p* < 0.01. (C) Relative mRNA expression of GAPDH in 293 T cells transfected with GAPDH 3′UTR-targeting siRNAs (siGAPDH 1, siGAPDH 2) or siRNA NC. Data are the means ± SD and *n* = 3 per group. Statistical significance was determined using one-way ANOVA followed by Dunnett’s multiple comparisons test *∗∗∗∗p* < 0.0001. (D) Immunoblot analysis of FLAG-tagged exogenous GAPDH(E) and GAPDH in 293 T cells. Knockdown of endogenous GAPDH with siRNA followed by the overexpression of GAPDH (E). Data are the means ± SD and *n* = 3 per group. Statistical significance was determined using one-way ANOVA followed by Dunnett’s multiple comparisons test with *∗p* < 0.05; ns, not significant. (E) Relative mRNA expression of *GAPDH* in 293 T cells knockdowned endogenous GAPDH (siGAPDH) and overexpressed GAPDH (C) and GAPDH (E), respectively. Data are the means ± SD and *n* = 3 per group. Statistical significance was determined using one-way ANOVA followed by Tukey’s multiple comparisons test with *∗∗p* < 0.01; *∗∗∗p* < 0.001; ns, not significant. (F) GAPDH activity assay in 293 T cells transfected with GAPDH 3′UTR siRNA, and overexpressing GAPDH(C), GAPDH(E). Data are the means ± SD and *n* = 4 per group. Statistical significance was determined using one-way ANOVA followed by Tukey’s multiple comparisons test with *∗p* < 0.05; *∗∗∗∗p* < 0.0001; ns, not significant. (G) Concentrations of lactate and pyruvate in 293 T cells transfected with GAPDH 3′UTR siRNA, and overexpressing GAPDH(C), GAPDH(E). Data are the means ± SD and *n* = 4 per group. Statistical significance was determined using one-way ANOVA followed by Tukey’s multiple comparisons test with *∗p* < 0.05, *∗∗p* < 0.01, and *∗∗∗∗p* < 0.0001. (H) Relative mRNA expression of *IL-6*, *TNF-α*, and *IL-1β* in THP-1 cells which transfected with GAPDH 3′UTR siRNA, and overexpressing GAPDH(C) or GAPDH(E). Data are the means ± SD and *n* = 3 per group. Statistical significance was determined using one-way ANOVA followed by Tukey’s multiple comparisons test with *∗p* < 0.05; *∗∗p* < 0.01; *∗∗∗p* < 0.001; ns, not significant. (I) The concentration of TNF-α in THP-1 cells that overexpressed GAPDH(C) or GAPDH(E). Data are the means ± SD and *n* = 3 per group. Statistical significance was determined using one-way ANOVA followed by Tukey’s multiple comparisons test with *∗∗∗p* < 0.001; *∗∗∗∗p* < 0.0001; ns, not significant.

Finally, to further investigate the effects of carboxyethylation modification of GAPDH on metabolism and inflammation, we further knocked down endogenous GAPDH and overexpressed exogenous mutated GAPDH. We knocked out GAPDH with 3′UTR-targeting siRNA and then overexpressed GAPDH(C) or GAPDH(E) (Figure 5A). The reduction of endogenous GAPDH in mRNA and protein levels was detected (Figures 5B and 5C). Subsequently, exogenous GAPDH(C) or GAPDH(E) was overexpressed. The immunoblot results showed that siGAPDH reduced the expression of endogenous GAPDH but did not affect the expression of exogenous GAPDH (Figure 5D). At the mRNA level, *GAPDH* mRNA levels were consistent in cells transfected with GAPDH(C) and GAPDH(E) plasmids (Figure 5E). After knocking down endogenous GAPDH, compared with GAPDH(C), the enzyme activity of GAPDH(E) decreased compared to GAPDH(C) (Figure 5F). Additionally, the content of pyruvate and lactate in cells was lower when GAPDH(E) was overexpressed (Figure 5G). Subsequently, THP-1 cells, which overexpress mutant GAPDH after knockdown of endogenous GAPDH, were stimulated with LPS for 24 h. *IL-6*, *TNF-α,* and *IL-1β* mRNA levels were decreased in cells overexpressing GAPDH(E) compared to GAPDH(C) (Figure 5H). In THP-1 cells, overexpression of GAPDH (E) resulted in a significant decrease in the level of inflammatory factor TNF-α compared to GAPDH(C) (Figure 5I). Furthermore, we constructed a GAPDH(A) plasmid encoding a GAPDH mutant that cannot be carboxyethylated by 3-HPA at position 247 due to site-directed mutagenesis. In cells where endogenous GAPDH was knocked down, and GAPDH(A) was overexpressed, 3-HPA treatment no longer affected GAPDH enzymatic activity, altered the cellular levels of pyruvate and lactate, and the mRNA levels of *IL-6, TNF-α*, and *IL-1β* (Figures S9A–S9D).

Collectively, these results indicated that 3-HPA induced GAPDH carboxyethylation, which promoted GAPDH degradation, reduced GAPDH enzyme activity, and switched macrophages from glycolysis to mitochondrial oxidation to alleviate the inflammatory response.

## Discussion

This study systematically describes a metabolic regulatory mechanism by which 3-HPA affects the function of macrophages. 3-HPA induces carboxyethylation of GAPDH at Cys247, triggering proteasomal degradation of GAPDH. This dual effect of reduced GAPDH protein abundance and enzymatic activity shifts macrophage metabolism from pro-inflammatory glycolysis to anti-inflammatory mitochondrial oxidation in macrophages, ultimately mitigating sepsis-associated inflammation.

The metabolic changes of immune cells become as important as the signaling pathways as the determinants of specific immune responses. A key consequence of these changes is the PTM of proteins by metabolites. A rich diversity of PTMs in macrophages has been identified, altering the phenotype to regulate immunity and inflammation in different contexts.^22^ We demonstrated that carboxyethylation of GAPDH at cysteine 247 affected the enzymatic activity and protein expression of GAPDH. The mechanism is that GAPDH carboxythylation affects protein stability and promotes protein degradation by the ubiquitin proteasome. It has been reported that mutating lysine at position 251 of GAPDH to glutamic acid also reduced the stability and enzymatic activity of GAPDH and increased GAPDH ubiquitination.^23^ These results suggest that a PTM near position 247 of GAPDH with a carboxyl group may affect the protein stability and enzyme activity of GAPDH. Subsequently, we conducted molecular dynamics simulations on the GAPDH protein and GAPDH (E) with cysteine at position 247 mutated to glutamic acid. The results indicated that the structural differences in the proteins originated from the unstable loop region at positions 183–207. GAPDH is composed of four subunits (O, P, Q, R), each of which binds to an NAD^+^ molecule. Each subunit consists of an N-terminal NAD^+^ binding domain (residues 1–150 and 314–335) and a C-terminal catalytic domain (residues 149–313).^24^ Furthermore, GAPDH 183-207 may have a D-glyceraldehyde 3-phosphate binding site. These results suggest that carboxyethylation modification at position 247 of GAPDH affects the catalytic domain of GAPDH, which in turn may affect function. Additionally, we observed that 3-HPA significantly decreases GAPDH protein expression while simultaneously increasing its mRNA transcription, which likely represents a compensatory transcriptional feedback mechanism in response to the accelerated degradation of the GAPDH protein. Given that 3-HPA induces GAPDH carboxyethylation and subsequent degradation, cells may sense the decline in this key glycolytic enzyme and activate transcriptional programs to restore protein homeostasis. As reported in the literature, feedback mechanisms exist in gene expression that allow for the upregulation or downregulation of protein production or degradation in response to steady-state protein levels.^25^

During LPS stimulation, macrophages undergo pseudo-hypoxia (also known as the Warburg effect) and switch their cellular metabolism from OXPHOS to anaerobic glycolysis.^3^ Using 2-DG in macrophages to block glucose uptake necessary for macrophage polarization reduces the LPS-stimulated production of inflammatory cytokines such as IL-1β.^26^ Our data suggested that the anti-inflammatory effect of 3-HPA is mediated in part by the inhibition of glycolysis. However, the anti-inflammatory effect of 3-HPA was partially maintained under hyperglycemic conditions or under conditions where glycolysis was inhibited by 2-DG, suggesting that there are other metabolic pathways that regulate macrophage function. In fact, our study found that 3-HPA increases TCA cycle metabolites and enhances mitochondrial oxidation. Our further analysis showed that the inhibition of GAPDH enzyme activity by 3-HPA resulted in an increase in NAD^+^/NADH content. Research reports that the NAD^+^-SIRT1-PGC-1α axis activates mitochondrial function and fatty acid oxidation genes (including CPT1A).^21^ We observed an increase in arginine metabolism, TCA cycle, and CPT1 enzyme transcription in macrophages treated with 3-HPA.

By demonstrating that 3-HPA shifts LPS-stimulated macrophages from glycolysis to mitochondrial oxidation, we validated that targeting metabolic switching is a viable strategy to reverse pro-inflammatory polarization. Notably, our results reinforce GAPDH as a critical node in this reprogramming: Previous studies have shown that GAPDH upregulation and enhanced glycolysis are defining features of activated pro-inflammatory macrophages,^17^ and our study extends this knowledge by identifying carboxyethylation targets GAPDH for degradation, complementing existing reports of GAPDH regulation through phosphorylation, nitrosylation, alkylation, succinylation, and malonylation modifications.^8^^,^^9^^,^^10^^,^^27^^,^^28^ Our research linked 3-HPA to innate immunity, demonstrating its role in the metabolic regulation of macrophages and supplementing the understanding of the immune function of 3-HPA. The key insight of our study lies in defining a complete molecular cascade: initiation by 3-HPA binding to GAPDH Cys247 and inducing carboxyethylation, effector steps where carboxyethylation promotes GAPDH K48 ubiquitination and proteasomal degradation to reduce GAPDH protein levels and enzymatic activity, metabolic switching as GAPDH inhibition elevates the NAD^+^/NADH ratio, increases arginine metabolism, TCA cycle and CPT1 enzyme transcription, and functional outcomes where mitochondrial oxidation enhancement shifts macrophages to an anti-inflammatory phenotype, reducing cytokine secretion and alleviating sepsis tissue damage. We speculate that 3-HPA treatment may have broader implications for other cell types beyond macrophages. For other immune cells, 3-HPA is likely to exert similar metabolic and functional modulation on T lymphocytes (naive and effector T cells rely on glycolysis for activation, proliferation, and cytokine secretion): 3-HPA-induced GAPDH Cys247 carboxyethylation would suppress glycolysis, elevate the NAD^+^/NADH ratio, and shift metabolism to mitochondrial oxidation, which may fine-tune T cell polarization. For neutrophil cells, which also undergo glycolysis-driven activation during inflammation, 3-HPA may similarly target GAPDH to restrain their pro-inflammatory effector functions via metabolic reprogramming. For non-immune cells with high glycolytic activity, such as cancer cells (driven by the Warburg effect) and intestinal epithelial cells (with high basal glycolysis), 3-HPA-mediated GAPDH inhibition may influence cancer cell proliferation and modulate intestinal epithelial energy metabolism, respectively.

In summary, this study identifies a mechanism by which 3-HPA modulates macrophage metabolism and inflammation: via GAPDH carboxyethylation, degradation, and subsequent glycolysis-to-mitochondrial oxidation switching. By linking a microbial metabolite to a specific PTM and metabolic pathway, our findings advance the understanding of metabolite-mediated immune regulation and provide a potential therapeutic target for sepsis and acute inflammation.

### Limitations of the study

Although our work reveals a regulatory role of GAPDH carboxyethylation in metabolic reprogramming, this study has several limitations. Immunofluorescence and cytoplasmic and nuclear separation experiments observed that carboxyethylated GAPDH was mainly located in the cytoplasm, suggesting that it may have regulatory effects in specific compartments. GAPDH-ce is confined in the cytoplasm and may prevent its non-metabolic function in the nucleus, which suggests that another layer of mechanism of immune regulation mediated by 3-HPA remains to be explored. Second, the current study focuses on the role of GAPDH carboxyethylation in macrophages, but other immune cells (e.g., neutrophils and T cells) also undergo metabolic reprogramming during sepsis. Whether 3-HPA exerts similar regulatory effects on these cell types remains unexplored. Investigating the impact of 3-HPA on the broader immune cell landscape will provide a more holistic understanding of its inflammation-regulatory mechanisms. Third, to directly establish the causal relationship between GAPDH carboxyethylation and enhanced oxidative phosphorylation in macrophages, future studies will require the generation of GAPDH C247E mutant mice to mimic this modification, followed by the assessment of glycolysis and oxidative phosphorylation in macrophages.

## Resource availability

### Lead contact

Further information and requests for resources and reagents should be directed to and will be fulfilled by the lead contact, Yue Zhai (zhaiyuefmmu@163.com).

### Materials availability

Plasmids and antibodies generated in this study will be made available on request, but we may require payment and/or a completed materials transfer agreement if there is potential for commercial application.

### Data and code availability

• The data supporting the findings of this study are available within the article and its supplementary materials. The RNA-seq data generated in this study have been deposited in the Gene Expression Omnibus (GEO) database. Accession numbers are listed in the key resources table.
• This paper does not report original code.
• Any additional information required to reanalyze the data reported in this paper is available from the lead contact upon request.

## Acknowledgments

This work was supported by the 10.13039/501100001809National Natural Science Foundation of China 82322029 (Y.Z.), 32541024 (Y.Z.), and 82471907 (H.-Y.L.), the Foundations of State Key Laboratory 20243BCC31007 (Y.Z.), the Shaanxi Provincial Youth Science and Technology New Star Program 2025ZC-KJXX-116 (H.-Y.L.), the Joint Innovation Foundation of Xijing Innovation Research Institute LHJJ24JH17 (Y.Z. and P.Z.), and the Cross-Integration Project 2023JC005 (P.Z.).

## Author contributions

Conception and design: K.-F.W., H.-Y.L., P.Z., and Y.Z.; development of methodology: K.-F.W., Y.-K.J., and Q.L.; data acquisition: K.-F.W., Y.-K.J., Q.L., F.W., T.-Y.L., M.-J.G., B.-D.G., Y.-M.Z., and S.-F.X.; analysis and data interpretation: K.-F.W., Y.-K.J., Q.L., D.S., L.L., H.-Y.L., and Y.Z.; writing, review, and/or revision of the manuscript: K.-F.W., H.-Y.L., and Y.Z; study supervision: H.-Y.L., P.Z., and Y.Z.

## Declaration of interests

Y.Z., P.Z., K.-F.W., Y.-M.Z, M.-J.G., and F.W. are inventors on patents applied for by Fourth Military Medical University related to GAPDH carboxyethylation.

## STAR★Methods

### Key resources table

**Table:** 

REAGENT or RESOURCE	SOURCE	IDENTIFIER
**Antibodies**		

GAPDH antibody	Proteintech	Cat# 60004-1-Ig, RRID:AB_2107436
Alpha Tubulin antibody	Proteintech	Cat# 66031-1-Ig; RRID:AB_11042766
Monoclonal ANTI-FLAG® M2 antibody	Sigma-Aldrich	Cat# F3165; RRID:AB_259529
Anti-ceC247 Antibody	This paper	N/A
Hexokinase 1 antibody	proteintech	Cat# 19662-1-AP; RRID:AB_10859778
PKM antibody	proteintech	Cat# 25659-1-AP; RRID:AB_2880181
LDHA-Specific antibody	proteintech	Cat# 19987-1-AP; RRID:AB_10646429
MYC-tag antibody	proteintech	Cat# 16286-1-AP; RRID:AB_11182162
Anti-ubiquitin Antibody	proteintech	Cat# 80992-1-RR; RRID:AB_2923694
Lamin B1 antibody	proteintech	Cat# 66095-1-Ig; RRID:AB_11232208
Anti-Ubiquitin (linkage-specific K48) antibody	Abcam	Cat# ab140601; RRID:AB_2783797
Goat anti-Rabbit IgG (H+L) Secondary Antibody, HRP	Thermo Fisher Scientific	Cat# 31460; RRID:AB_228341
Goat anti-Mouse IgG (H+L) Secondary Antibody, HRP	Thermo Fisher Scientific	Cat# 31430; RRID:AB_228307
mouse IgG	Beyotime	Cat# A7028, RRID:AB_2909433
Anti-GAPDH Antibody [SA30-01]	HUABio	Cat# ET1601-4, RRID:AB_3069615
Donkey anti-Rabbit IgG (H+L) Highly Cross-Adsorbed Secondary Antibody, Alexa Fluor™ 488	Thermo Fisher Scientific	Cat# A-21206, RRID:AB_2535792
Donkey anti-Mouse IgG (H+L) Highly Cross-Adsorbed Secondary Antibody, Alexa Fluor™ 555	Thermo Fisher Scientific	Cat# A-31570, RRID:AB_2536180

**Bacterial and virus strains**		

Trelief® 5α Chemically Competent Cell	Tsingke	Cat# TSC-C01

**Chemicals, peptides, and recombinant proteins**		

3-Hydroxypropanoic acid	Macklin	Cat# H823102
MG132	MCE	Cat# HY-13259
Leupeptin	MCE	Cat# HY-18234 A
Bafilomycin A1	MCE	Cat# HY-100558
Cycloheximide (CHX)	MCE	Cat# HY-12320
Phorbol-12-myristate-13-acetate (PMA)	MCE	Cat# HY-18739
Recombinant mouse M-CSF (carrier-free)	BioLegend	576404
LPS	Beyotime	ST1470
Lipofectamine™ 2000 Transfection Reagent	Thermo Invitrogen	Cat# 11668019
Lipopolysaccharides from E. coli O111:B4	SIGMA	Cat# L2630
TMB (high sensitivity)	NeoBioscience	Cat# TMH 600
TRIzol reagent	Thermo Fisher Scientific	Cat#15596026
Loading Buffer(5x)	Beyotime	Cat#P0015
RIPA buffer	Beyotime	Cat#P0013B
DAPI	Beyotime	Cat#C1002

**Critical commercial assays**		

BCA Protein Assay Kit	Beyotime	P0011
Lactic Acid assay kit	Nanjing Jiancheng Bioengineering Institute	A019-2-1
Pyruvate Assay Kit	Nanjing Jiancheng Bioengineering Institute	A081-1-1
Enhanced ATP Assay Kit	Beyotime	S0027
GAPDH Activity Assay Kit	Abcam	ab204732
Mitochondrial Membrane Potential Assay Kit (TMRE)	Beyotime	C2001S
Enhanced NAD^+^/NADH Assay Kit with WST-8	Beyotime	S0176S
Nuclear and Cytoplasmic Protein Extraction Kit	Beyotime	P0028
Mouse TNF-α Precoated ELISA Kit	Dakewe	1217202
Mouse IL-6 Precoated ELISA Kit	Dakewe	1210602
Mouse IL-1β Precoated ELISA Kit	Dakewe	1210122
Human TNF-α Precoated ELISA Kit	Dakewe	1117202
Human IL-6 Precoated ELISA Kit	Dakewe	1110602
Human IL-1β Precoated ELISA Kit	Dakewe	1110122
TB Green® Premix Ex Taq™ II (Tli RNase H Plus)	TAKARA	RR820A
PrimeScript™ RT Master Mix (Perfect Real Time)	TAKARA	RR036A
Total RNA Kit Ⅱ	Omega	R6934-01

**Deposited data**		

RNA-seq data	This study	GEO: GSE316690

**Experimental models: Cell lines**		

Human: THP-1	ATCC	Cat# TIB-202
Human: HEK293T cell	ATCC	Cat# CRL-11268
Mouse: primary BMDMs	This study	N/A

**Experimental models: Organisms/strains**		

Mouse: C57BL/6 J	Tengxin	N/A

**Oligonucleotides**		

siGAPDH1 for human GAPDH sense (5′-3′) CAUCAAUAAAGUACCCUGUTT	This study	N/A
siGAPDH1 for human GAPDH antisense (5′-3′) ACAGGGUACUUUAUUGAUGTT	This study	N/A
siGAPDH2 for human GAPDH sense (5′-3′) UGCCACACUCAGUCCCCCATT	This study	N/A
siGAPDH2 for human GAPDH antisense (5′-3′) UGGGGGACUGAGUGUGGCATT	This study	N/A
*Il1-β*, Mouse, Forward Primer (5′-3′) TGGCAACTGTTCCTG,	This study	N/A
*Il1-β*, Mouse, Reverse Primer (5′-3′) GGAAGCAGCCCTTCATCTTT	This study	N/A
*Il-6*, Mouse, Forward Primer (5′-3′) CTGCAAGAGACTTCCATCCAG	This study	N/A
*Il-6*, Mouse, Reverse Primer (5′-3′) AGTGGTATAGACAGGTCTGTTGG	This study	N/A
*Tnf-α*, Mouse, Forward Primer (5′-3′) CAGGCGGTGCCTATGTCTC	This study	N/A
*Tnf-α*, Mouse, Reverse Primer (5′-3′) CGATCACCCCGAAGTTCAGTAG	This study	N/A
*Actin*, Mouse, Forward Primer (5′-3′) GGCTGTATTCCCCTCCATCG	This study	N/A
A*ctin*, Mouse, Reverse Primer (5′-3′) CCAGTTGGTAACAATGCCATGT	This study	N/A
*GAPDH*, Human, Forward Primer (5′-3′) GCACCGTCAAGGCTGAGAAC	This study	N/A
*GAPDH*, Human, Reverse Primer (5′-3′) TGGTGAAGACGCCAGTGGA	This study	N/A
*ACTIN*, Human, Forward Primer (5′-3′) CACCATTGGCAATGAGCGGTTC	This study	N/A
*ACTIN*, Human, Reverse Primer (5′-3′) AGGTCTTTGCGGATGTCCACGT;	This study	N/A
*CPT1A*, Human, Forward Primer (5′-3′) TCCAGTTGGCTTATCGTGGTG	This study	N/A
*CPT1A*, Human, Reverse Primer (5′-3′) TCCAGAGTCCGATTGATTTTTGC	This study	N/A
*CPT1B*, Human, Forward Primer (5′-3′) GCGCCCCTTGTTGGATGAT	This study	N/A
*CPT1B*, Human, Reverse Primer (5′-3′) CCACCATGACTTGAGCACCAG	This study	N/A
*Cpt1a*, Mouse, Forward Primer (5′-3′) CTCCGCCTGAGCCATGAAG	This study	N/A
*Cpt1a*, Mouse, Reverse Primer (5′-3′) CACCAGTGATGATGCCATTCT	This study	N/A
*Cpt1b*, Mouse, Forward Primer (5′-3′) GCACACCAGGCAGTAGCTTT	This study	N/A
*Cpt1b*, Mouse, Reverse Primer (5′-3′) CAGGAGTTGATTCCAGACAGGTA	This study	N/A
*Il-10*, Mouse, Forward Primer (5′-3′) GCCCAGAAATCAAGGAGCATTTG	This study	N/A
*Il-10*, Mouse, Reverse Primer (5′-3′) ACACCTTGGTCTTGGAGCTTATT	This study	N/A
*Tgfb1*, Mouse, Forward Primer (5′-3′) GCAACAACGCCATCTATGAGAAA	This study	N/A
*Tgfb1*, Mouse, Reverse Primer (5′-3′) AACGCCAGGAATTGTTGCTATAT	This study	N/A
*Arg-1*, Mouse, Forward Primer (5′-3′) TGGCCTTTGTTGATGTCCCTAAT	This study	N/A
*Arg-1*, Mouse, Reverse Primer (5′-3′) GCATCCACCCAAATGACACATAG	This study	N/A
*IL-10*, Human, Forward Primer (5′-3′) GCCTTGTCTGAGATGATCCAGTT	This study	N/A
*IL-10*, Human, Reverse Primer (5′-3′) TCACAGGGAAGAAATCGATGACA	This study	N/A
*TGFB1*, Human, Forward Primer (5′-3′) TACAGCAACAATTCCTGGCGATA	This study	N/A
*TGFB1*, Human , Reverse Primer (5′-3′) CAGTGTGTTATCCCTGCTGTCAC	This study	N/A
*ARG-1*, Human, Forward Primer (5′-3′) GTGGAAGAAGGCCCTACAGTATT	This study	N/A
*ARG-1*, Human, Reverse Primer (5′-3′) AGGCTGATTCTTCCGTTCTTCTT	This study	N/A

**Recombinant DNA**		

Human GAPDH ORF mammalian expression plasmid, C-Flag tag	sino Biological	HG10094-CF
Human GAPDH C247D ORF mammalian expression plasmid, C-Flag tag	This study	N/A
Human GAPDH C247E ORF mammalian expression plasmid, C-Flag tag	This study	N/A
Human GAPDH C247M ORF mammalian expression plasmid, C-Flag tag	This study	N/A
pcDNA3.1-Ub-K6O, Myc tag	This study	N/A
pcDNA3.1-Ub-K11O, Myc tag	This study	N/A
pcDNA3.1-Ub-K27O, Myc tag	This study	N/A
pcDNA3.1-Ub-K29O, Myc tag	This study	N/A
pcDNA3.1-Ub-K33O, Myc tag	This study	N/A
pcDNA3.1-Ub-K48O, Myc tag	This study	N/A
pcDNA3.1-Ub-K63O, Myc tag	This study	N/A
pcDNA3.1-Ub, Myc tag	This study	N/A

**Software and algorithms**		

ImageJ	NIH	https://imagej.nih.gov/ij/
GraphPad Prism 10	GraphPad Software Inc.	https://www.graphpad.com/
Pymol	Schrodinger	https://pymol.org/2/
Seahorse XFe 96 Wave software (v2.6)	Agilent	https://www.agilent.com
Image Lab software	Bio-Rad	https://www.bio-rad.com
TBtools	Chengjie Chen et al., 2020	https://github.com/CJ-Chen/TBtools

### Experimental model and study participant details

#### Animals and treatments

##### Mice

Specific pathogen-free (SPF) grade C57BL/6 N mice (6–8 weeks old, male) were purchased from Chongqing Tengxin Biotechnology Co., LTD and were housed under a 12-h light/12-h dark cycle at a constant temperature (22°C) with free access to food and water. All animal experiments were approved by the Institutional Animal Care and Use Committee (IACUC) of the Laboratory Animal Center, Fourth Military Medical University (20230174). Regarding the influence of sex: only male mice were used in this study. As no cross sex comparative analyses were performed, the potential impact of sex on the reported outcomes remains undetermined, which represents a limitation regarding the generalizability of the conclusions.

##### LPS-induced bacteremia model

Mice were randomly divided into three groups: control group, LPS group, and LPS+3-HPA group. Mice in the LPS group and LPS+3-HPA group were intraperitoneally injected with lipopolysaccharide (LPS, Escherichia coli O111:B4, Sigma-Aldrich) at a dose of 5 mg/kg to induce systemic inflammatory response. Mice in the LPS+3-HPA group were intraperitoneally injected with 3-hydroxypropanoic acid (3-HPA, Macklin) at a dose of 50 mg/kg 2 h before LPS administration; mice in the control group were injected with an equal volume of physiological saline. At 8 h after LPS injection, mice were euthanized, and lung and liver tissues were collected for histological analysis and RNA extraction.

##### Cecal ligation and perforation (CLP)-Induced sepsis model

Mice were anesthetized with 10% chloral hydrate (350 mg/kg, intraperitoneal injection). A midline abdominal incision (1–2 cm) was made to expose the cecum. The cecum was ligated with 4-0 silk suture at 1 cm from the cecum tip, and a 25-gauge needle was used to perforate the ligated cecum twice. A small amount of fecal content was extruded to ensure patency of the perforation, and the cecum was returned to the abdominal cavity. The abdominal wall was sutured layer by layer. Mice were injected with 100 μl physiological saline. Mice in the CLP+3-HPA group were intraperitoneally injected with 3-HPA (50 mg/kg) immediately after surgery and once every 24 h thereafter; mice in the CLP group and control group were injected with an equal volume of physiological saline. Four days after surgery, mice were euthanized for subsequent experiments.

#### Cell culture and treatment

##### Cell lines

The THP-1 and HEK 293 T (293 T) cell lines purchased from ATCC were cultured in RPMI-1640 medium supplemented with 10% fetal bovine serum (FBS), 1% penicillin–streptomycin, and 1% L-glutamine at 37°C in a humidified atmosphere with 5% CO_2_. All cell lines used were authenticated via STR genotyping and tested negative for mycoplasma contamination.

##### Isolation and culture of bone marrow-derived macrophages (BMDMs)

Bone marrow cells were isolated from the femurs and tibias of C57BL/6 N mice. After removing red blood cells with red blood cell lysis buffer (TIANGEN), cells were resuspended in RPMI 1640 medium containing 10% fetal bovine serum (FBS, Corning), 1% penicillin-streptomycin, 1% L-glutamine and 20 ng/mL macrophage colony stimulating factor (M-CSF, Biolegend). Cells were cultured in a 37°C, 5% CO_2_ incubator, and the medium was changed every 3 days. After 7 days of culture, adherent cells were identified as BMDMs by flow cytometry (CD11b^+^F4/80^+^) and used for subsequent experiments.

##### Culture and differentiation of THP-1 cells

Human monocytic THP-1 cells (ATCC) were cultured in RPMI 1640 medium containing 10% FBS, 1% penicillin-streptomycin, and 1% L-glutamine at 37°C, 5% CO_2_. To induce differentiation into macrophages, THP-1 cells were seeded in 6-well plates (1×10^6^ cells/well) and treated with 200 ng/mL phorbol 12-myristate 13-acetate (PMA, MCE) for 24 h. After differentiation, the medium was replaced with fresh RPMI 1640 medium without PMA, and cells were cultured for another 24 h before treatment.

##### Cell treatment

THP-1 cells were divided into PBS group, LPS group, and LPS+3-HPA group. Cells in the LPS group and LPS+3-HPA group were stimulated with 100 ng/mL LPS; Cells in the LPS+3-HPA group were treated simultaneously with 100 ng/mL LPS and 5 mM 3-HPA. Cells in the PBS group were treated with an equal volume of medium. After 24 h of LPS stimulation, cell supernatants were collected for ELISA, and cells were collected for RNA extraction, protein extraction, or metabolomic analysis.

BMDMs were divided into PBS group, LPS group, and LPS+3-HPA group. Cells in the LPS group and LPS+3-HPA group were stimulated with 100 ng/mL LPS; Cells in the LPS+3-HPA group were treated simultaneously with 100 ng/mL LPS and 5 mM 3-HPA. Cells in the control group were treated with an equal volume of medium. After 24 h of LPS stimulation, cell supernatants were collected for ELISA, and cells were collected for RNA extraction, protein extraction, or metabolomic analysis.

### Method details

#### Enzyme-linked Immunosorbent assay (ELISA)

The concentrations of IL-6, IL-1β and TNF-α in cell supernatants and mouse serum were detected using mouse IL-6 ELISA kit, mouse IL-1β ELISA kit, mouse TNF-α ELISA kit, human IL-6 ELISA kit, human TNF-α ELISA kit and human IL-1β ELISA kit (all from Dakewei) according to the manufacturer’s instructions. Briefly, 100 μL of standard or sample was added to each well of the ELISA plate, and add 50 μL the Biotinylated Antibody working solution to each well. After mixing well, cover with a sealing film and incubate at room temperature (18°C–25°C) for 2 h.

And then the plate was washed 3 times with washing buffer. 100 μL of streptavidin-horseradish peroxidase (HRP) was added to each well, incubated at 37°C for 1 h, and washed 3 times. Finally, 100 μL of substrate solution (tetramethylbenzidine, TMB) was added, incubated at 37°C for 15 min in the dark, and 100 μL of stop solution was added. The absorbance at 450 nm was measured using a microplate reader, and the concentration of the target cytokine was calculated based on the standard curve.

#### Histological analysis

Lung and liver tissues were fixed in 4% paraformaldehyde (Boster), dehydrated with gradient ethanol, embedded in paraffin, and cut into 5-μm-thick sections by Shaanxi Yike biotechnology Service Co., Ltd. Sections were stained with hematoxylin and eosin according to standard procedures. Stained sections were observed under a light microscope and histological changes were evaluated in a double-blind manner. The severity of lung damage was evaluated based on alveolar hemorrhage, interstitial inflammation, and hyaline membrane formation; the severity of liver damage was evaluated based on hepatocyte swelling, necrosis, and inflammatory cell infiltration.

#### RNA extraction and quantitative Real-Time PCR (qRT-PCR)

Total RNA was extracted from tissues or cells using TRK Lysis Buffer (Omega) according to the manufacturer’s instructions. The concentration and purity of RNA were detected using a NanoDrop 2000 spectrophotometer (Thermo Fisher Scientific). cDNA was synthesized from 1 μg of total RNA using a PrimeScript RT Reagent Kit with gDNA Eraser (TaKaRa). qRT-PCR was performed using an SYBR Premix Ex Taq II Kit (TaKaRa) on a Real-Time PCR System (Bio-Rad). The relative expression level of target genes was calculated using the 2^-ΔΔCt^ method, with actin as the internal reference gene. All primers were synthesized by Beijing Tsingke Biotech Company.

#### RNA sequencing (RNA-seq) and bioinformatics analysis

RNA-Seq was performed by Obio Technology (Shanghai) Co.,Ltd. Total RNA was extracted from THP-1 cells (with or without 3-HPA treatment) using TRIzol reagent and sent to Obio Technology. Gene Ontology (GO) enrichment analysis and Kyoto Encyclopedia of Genes and Genomes (KEGG) pathway enrichment analysis of DEGs were performed using clusterProfiler software.

#### Protein extraction and immunoblotting

Cells or tissues were lysed in RIPA lysis buffer (Beyotime) containing 1% phenylmethylsulfonyl fluoride (PMSF, Beyotime) on ice for 30min. The lysate was centrifuged at 16,000×g for 20 min at 4°C, and the supernatant was collected as total protein. The protein concentration was determined using a BCA Protein Assay Kit (Beyotime).

Equal amounts of protein were separated by 10% sodium dodecyl sulfate-polyacrylamide gel electrophoresis (SDS-PAGE) and transferred to polyvinylidene fluoride (PVDF) membranes (Millipore). Membranes were blocked with 5% non-fat milk (Solarbio) for 1 h at room temperature, and then incubated with primary antibodies overnight at 4°C. After washing with phosphate buffered saline with Tween 20 (PBST) three times (10min each), membranes were incubated with the HRP-conjugated anti-mouse IgG or anti-rabbit IgG secondary antibodies (Thermo Fisher Scientific) for 1 h at room temperature. Membranes were washed again with PBST three times, and protein bands were visualized using an enhanced chemiluminescence (ECL) detection kit (Epizyme). The gray value of protein bands was quantified using ImageJ software.

#### Preparation and validation of Anti-ceC247 specific monoclonal antibody

GAPDH-ceC247 Specific Monoclonal Antibody was developed in our laboratory as follows.

First, a synthetic peptide corresponding to the amino acid sequence of mouse GAPDH around the Cys247 residue (VVDLTCceRLEKPAKYC-KLH) was used as the immunogen. This peptide (purity ≥95%) was activated by maleimide and coupled with KLH, and was synthesized and verified by Chinese Peptide Company. Then, six-week-old male SPF-grade BALB/C mice were immunized: for primary immunization, 100 μg/mouse of KLH-conjugated peptide emulsified with CFA was injected subcutaneously at 4–6 back sites; two weeks later, 3 booster immunizations were performed every 2 weeks with 50 μg/mouse of KLH-conjugated peptide emulsified with IFA. Three days before cell fusion, a final intravenous boost of 100 μg/mouse of KLH-conjugated peptide (dissolved in sterile saline) was given via intraperitoneal injection. Next, cell fusion and hybridoma screening were conducted: spleen cells from mice with the highest serum antibody titer were isolated, lysed of red blood cells, and mixed with logarithmic-phase SP2/0 myeloma cells at a 5:1 ratio; fusion was induced with 1 mL of pre-warmed PEG 1500, terminated with RPMI 1640 medium, and the fused cells were resuspended in HAT medium and seeded into 96-well plates (100 μL/well) for culture (half the medium replaced every 3 days); When hybridoma colonies appeared, indirect ELISA was used for primary screening (coating carboxyethylated and unmodified peptides, detecting with hybridoma supernatant, positive if OD_450_ of carboxyethylated peptide ≥2.1 times that of unmodified peptide and negative control). Positive hybridomas were subcloned 2–3 times by limiting dilution to obtain monoclonal hybridoma cell lines. For antibody production and purification: Collect the supernatant of monoclonal hybridoma cells, centrifuge to remove debris, and purify the antibody using a HiTrap Protein An HP antibody purification column.

#### ELISA validation

The affinity and specificity of the anti-ceC247 antibody were detected by ELISA. Briefly, the carboxyethylated GAPDH Cys247 peptide and unmodified GAPDH Cys247 peptide were coated onto ELISA plates (1 μg/well) overnight at 4°C. Plates were blocked with 5% non-fat milk for 1 h, and then incubated with the purified anti-ceC247 antibody for 1 h at 37°C. After washing 3 times, HRP-conjugated anti-mouse IgG secondary antibody was added for 1 h at 37°C. After washing three times, 100 μL of TMB was added, incubated at 37°C for 15 min in the dark, and 50 μL of stop solution was added. The absorbance at 450 nm was measured using a microplate reader. The anti-ceC247 antibody was considered specific if it showed a significantly higher binding signal to the carboxyethylated peptide than to the unmodified peptide.

#### Surface plasmon resonance (SPR) detection

SPR analysis was performed using a Biacore T200 instrument (GE Healthcare) to determine the affinity of the anti-ceC247 antibody for the carboxyethylated GAPDH Cys247 peptide. The anti-ceC247 antibody was immobilized on a CM5 sensor chip using the amine coupling method. The carboxyethylated GAPDH Cys247 peptide and unmodified GAPDH Cys247 peptide were serially diluted and injected over the chip surface at a flow rate of 30 μL/min. The association and dissociation phases were recorded, and the affinity constant (KD) was calculated using Biacore T200 Evaluation Software.

#### Dot-blot validation

The carboxyethylated GAPDH Cys247 peptide, unmodified GAPDH Cys247 peptide, and lactated GAPDH Cys247 peptide were spotted onto nitrocellulose (NC) membranes (Millipore) and dried at room temperature. Membranes were blocked with 5% non-fat milk for 1 h, incubated with the anti-ceC247 antibody (1:1000 dilution) at 37°C for 2 h. After washing three times, then incubated with HRP-labeled secondary antibody at 37°C for 1 h. The signal was visualized using ECL, and the specificity of the anti-ceC247 antibody was confirmed.

#### Plasmid construction and cell transfection

The GAPDH Human Plasmid Kit was purchased from Origene. Site-directed mutagenation was accomplished by AUGCT Biotechnology Co., Ltd. to generate mutant plasmids: GAPDH-D (Cys247→Asp), GAPDH-E (Cys247→Glu), and GAPDH-M (Cys247→Met). All plasmids were verified by DNA sequencing.

293 T cells were cultured in RPMI 1640 medium containing 10% FBS, 1% penicillin-streptomycin, and 1% L-glutamine at 37°C, 5% CO_2_. Cells were seeded in 6-well plates (2×10^5^ cells/well) 24 h before transfection. Transfection was performed using Lipofectamine 2000 reagent (Thermo Fisher Scientific) according to the manufacturer’s instructions. For transient transfection, 2 μg of plasmid DNA was used per well. The expression of GAPDH (wild-type and mutants) was detected by immunoblotting.

#### Protein stability assay

293 T cells transfected with GAPDH-C, GAPDH-D, GAPDH-E, GAPDH-M were treated with 2 μg/mL cycloheximide (CHX, MCE) to inhibit protein synthesis. Cells were collected at 0 h, 12 h, and 24 h after CHX treatment, and total protein was extracted. The expression level of GAPDH was detected by immunoblotting.

To determine the degradation pathway, cells were treated with 5 μM MG132 (MCE), 100 μM Leupeptin (MCE), 5 nM bafilomycin A1 (MCE), or 50 μM chloroquine (MCE) for 24 h. The stability of GAPDH was analyzed by immunoblotting as described above.

#### Co-immunoprecipitation (Co-IP)

293 T cells were transfected with GAPDH-WT, and Myc-Ub (or its mutants) for 48 h. Cells were lysed in IP lysis buffer (Thermo Fisher Scientific) containing 1% PMSF and 1% protease inhibitor cocktail. The lysate was centrifuged at 16,000×g for 20 min at 4°C, and the supernatant was collected. GAPDH or anti-ceC247 specific antibodies and protein samples from cell lysates were used for IP and CO-IP assays according to the manufacturer’s protocol (Thermo Scientific).

#### Immunofluorescence staining

THP-1 cells were seeded into DISH and subsequently subjected to PMA treatment for 24 h, resting, and LPS treatment for 24 h. Cells were washed twice with PBS, fixed with 4% paraformaldehyde for 15 min at room temperature, and then washed 3 times with PBS. Cells were permeabilized with 0.3% Triton X-100 for 10 min at room temperature, followed by blocking with 5% BSA for 1 h at 37°C. Subsequently, cells were incubated overnight at 4°C with primary antibodies: rabbit anti-GAPDH polyclonal antibody (R1210-1, HUABIO, 1:200 dilution) and mouse anti-ceC247 monoclonal antibody (1:200 dilution). The next day, cells were washed 3 times with PBST, then incubated with fluorochrome-conjugated secondary antibodies (Alexa Fluor 488-conjugated goat anti-rabbit IgG and Alexa Fluor 555-conjugated goat anti-mouse IgG, both at 1:1000 dilution) for 1 h at room temperature in the dark. After 3 additional washes with PBST, cell nuclei were stained with 4′,6-diamidino-2-phenylindole (DAPI, 1:1000 dilution) for 10 min. Images were visualized under a confocal fluorescence microscope (Nikon) to observe the subcellular localization of GAPDH and GAPDH-ce.

#### Nuclear and cytoplasmic fractionation

Nuclear and cytoplasmic fractionation of THP-1 cells was performed using the Nuclear and Cytoplasmic Protein Extraction Kit (Beyotime) following the manufacturer’s instructions. The protein concentration of both extracts was determined by BCA, and the extracts were used for subsequent immunoblotting to detect the distribution of GAPDH and GAPDH-ce in the nucleus and cytoplasm.

#### Detection of GAPDH enzyme activity

The activity of GAPDH in LPS-stimulated BMDMs and THP-1 cells (with or without 3-HPA treatment) was detected using the GAPDH Activity Assay Kit (Abcam) in strict accordance with the manufacturer’s instructions. And the absorbance value related to GAPDH activity was measured by a microplate reader to calculate the relative activity of GAPDH.

#### Detection of lactate and pyruvate levels

The levels of glycolytic metabolites lactate and pyruvate in LPS-stimulated BMDMs and THP-1 cells (with or without 3-HPA treatment) were detected using the Lactic Acid assay kit and Pyruvate Assay Kit (Nanjing Jiancheng Bioengineering Institute) following the manufacturer’s instructions.

#### Extracellular acidification rate (ECAR) and oxygen consumption rate (OCR)

ECAR and OCR were measured using a Seahorse XF96 Extracellular Flux Analyzer (Agilent Technologies). To examine glycolysis rates, the Seahorse Glycolysis Stress Assay was performed using the XF96 Extracellular Flux Analyzer. To examine mitochondrial function, the Seahorse Mitochondrial Stress Assay was performed using the XF96 Extracellular Flux Analyzer. BMDMs were seeded in Seahorse XF96 cell culture plates (1×10^5^ cells/well) and treated with LPS (10 ng/mL) and/or 3-HPA (5 mM) for 24 h. ECAR and OCR were measured according to the manufacturer’s protocol, and the data were analyzed using Seahorse XFe96 Analyser (Agilent).

#### Detection of mitochondrial membrane potential

Mitochondrial membrane potential (ΔΨm) was assessed using a TMRE (tetramethylrhodamine, ethyl ester) Mitochondrial Membrane Potential Assay Kit (Beyotime) according to the manufacturer’s instructions. Following treatment, THP-1 cells or bone marrow-derived macrophages (BMDMs) were harvested and resuspended in TMRE staining working solution at a density of approximately 1×10^6^ cells/mL. The working solution was prepared by diluting the TMRE (1000×) stock solution with the provided assay buffer at a ratio of 1:1000. Cells were then incubated at 37°C for 15–45 min in the dark. As a positive control for mitochondrial depolarization, cells were treated with 10 μM CCCP (carbonyl cyanide *m*-chlorophenyl hydrazone) for 20 min prior to TMRE staining. Following incubation, cells were washed twice with pre-warmed cell culture medium by centrifugation at 400×*g* for 5 min at room temperature. The cell pellet was resuspended in fresh medium, and TMRE fluorescence was analyzed using a flow cytometer with excitation at 550 nm and emission at 575 nm.

#### Detection of NAD^+^ and NADH levels

The levels of NAD^+^ and NADH in BMDMs and THP-1 cells were detected using the enhanced NAD^+/^NADH Detection Kit (Beyotime) according to the manufacturer’s instructions. Briefly, cells were lysed in NAD^+^/NADH extraction buffer, and the lysate was centrifuged at 12,000×g for 5 min at 4°C. The supernatant was divided into two parts: one part was used to detect total NAD (NAD^+^+NADH) after heat treatment (60°C for 30 min to decompose NADH), and the other part was used to detect NADH. The reaction mixture containing the sample, assay buffer, and enzyme mix was incubated at 37°C for 30 min, and the absorbance at 450 nm was measured. The concentrations of NAD^+^ and NADH were calculated based on standard curves, and the NAD^+^/NADH ratio was calculated.

#### ATP level detection

The ATP level in LPS-stimulated BMDMs and THP-1 cells (with or without 3-HPA treatment) was detected using the Enhanced ATP Assay Kit (Beyotime) strictly following the manufacturer’s instructions. The luminescence intensity of each well was measured using a microplate reader with the luminescence detection mode. The ATP concentration in the sample was calculated based on the standard curve.

#### Metabolomic analysis

Targeted metabolomic analysis of metabolites in LPS-stimulated BMDMs (with or without 3-HPA) was conducted by Metabo-Profile Biotechnology Co., Ltd. High-performance liquid chromatography-tandem mass spectrometry (HPLC-MS/MS) was used for separation and detection. Metabo-Profile Biotechnology Co., Ltd. performed raw data filtering, metabolite quantification via external standard curves, and data normalization to cell protein concentration, with quality control samples used to ensure detection stability.

#### Transmission electron microscopy

BMDMs were fixed in 4% glutaraldehyde over 24 h. Ultrathin sections (70 nm) were cut, stained with uranyl acetate and lead citrate, and observed under a transmission electron microscope (HITACHI HT7800). The morphological changes of mitochondria were evaluated by two observers in a double-blind manner.

### Quantification and statistical analysis

All the biological experiments were repeated at least three times to assess reproducibility. The presented data were collected from biologically independent samples. All data are expressed as the mean ± standard deviationn (SD). The differences between the two groups were analyzed using unpaired two-tailed Student’s *t* test, and for multiple-group experiments, one-way ANOVA was used followed by Tukey’s or Dunnett’s multiple comparisons test. The analysis was performed using GraphPad Prism. *p* < 0.05 was considered statistically significant, as indicated by asterisks in the figure legends. ns, not significant; *∗∗∗∗p* < 0.0001; *∗∗∗p* < 0.001; *∗∗p* < 0.01; *∗p* < 0.05. All statistical details of the experiments can be found in the figure legends.
