# Supplementary material for: 3-Hydroxypropionic acid converts inflammatory macrophage glycolysis into mitochondrial oxidation through GAPDH carboxyethylation
Source: iScience. 2026 Jun 4;29(6):116258. doi: 10.1016/j.isci.2026.116258 (PMC13266029; doi:10.1016/j.isci.2026.116258)
Supplement: Document S1. Figures S1–S9 [file mmc1.pdf]

iScience, Volume *29*

Supplemental information

3-Hydroxypropionic acid converts inflammatory macrophage glycolysis into mitochondrial oxidation through GAPDH carboxyethylation

Kefei Wu, Yankun Jia, Qi Lin, Fu Wang, Tianyue Liu, Mengjie Gao, Baodong Gao, Yumeng Zhu, Sifeng Xiong, Dong Sun, Ling Li, Huanyu Lu, Ping Zhu, and Yue Zhai

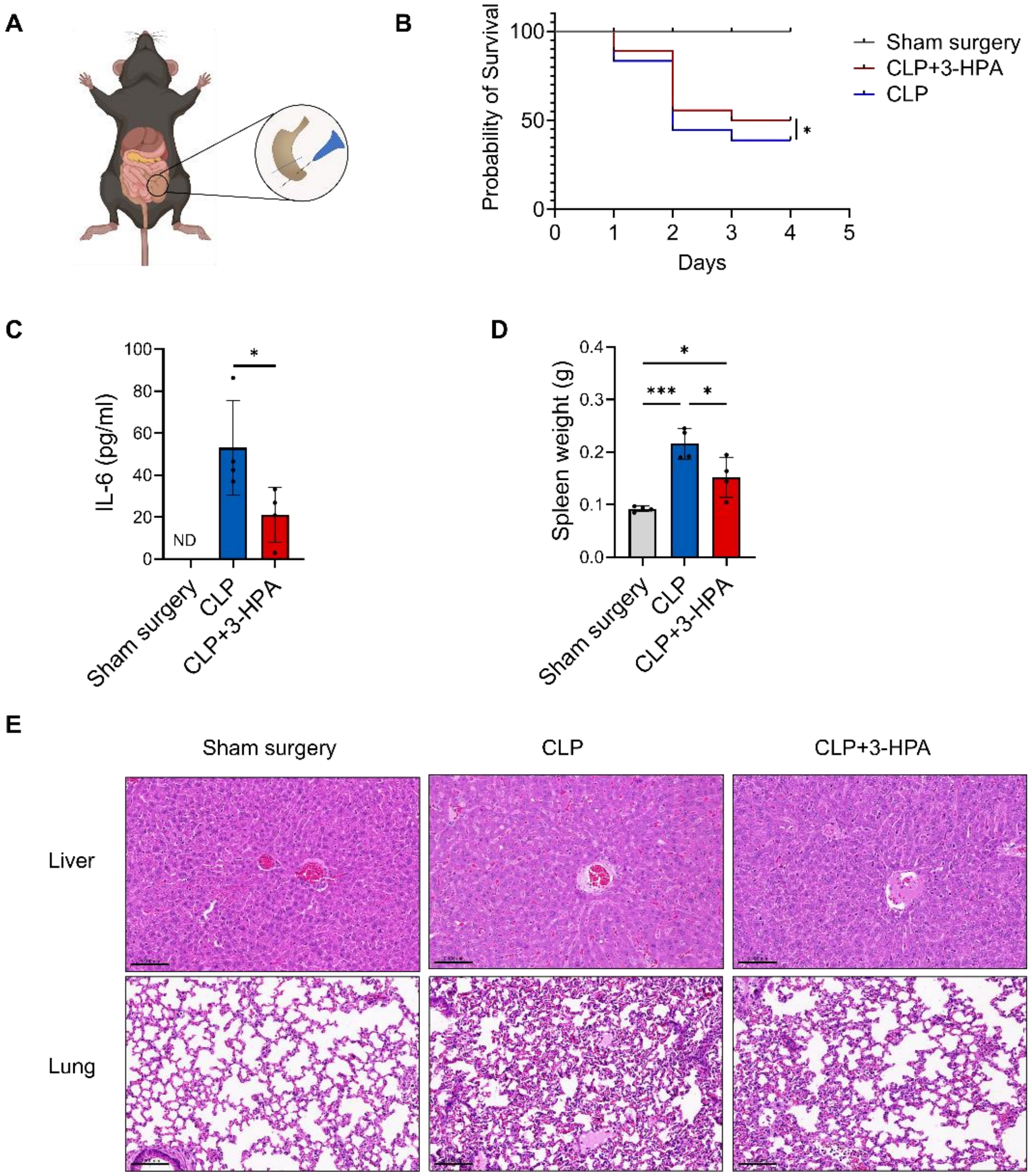

Fig S1. 3-HPA alleviates sepsis in the CLP model

(A) Schematic illustration of the CLP procedure for establishing a murine sepsis model. Created with BioRender.com.

(B) Kaplan-Meier survival curves of mice in sham surgery, CLP, or CLP with 3-HPA treatment group (50 mg/kg) over 4 days observation period. Log-rank (Mantel–Cox) test was used to compare the differences in survival rates between groups, with $*P<0.05$ considered statistically significant.

(C) Serum IL-6 concentrations in mice subjected to sham surgery, CLP, or CLP with 3-HPA treatment (50 mg/kg) were quantified by ELISA (n=4 per group). Data are the means ± SD and n=4 per group. Statistical significance was determined using unpaired Student's t test with $*P<0.05$. “ND" indicated "not detected".

(D) The spleen weight of mice subjected to sham surgery, CLP, or CLP plus 3-HPA treatment (50 mg/kg) were quantified (n=4 per group). Data are the means ± SD and n=4 per group.

Statistical significance was determined using one-way ANOVA followed by Tukey's multiple comparisons test with *$P<0.05$; **$P<0.01$; ***$P<0.001$.

(E) Representative HE (hematoxylin-eosin) staining of liver and lung tissues from sham surgery, CLP, and CLP+3-HPA groups (scale bar: 100 μm).

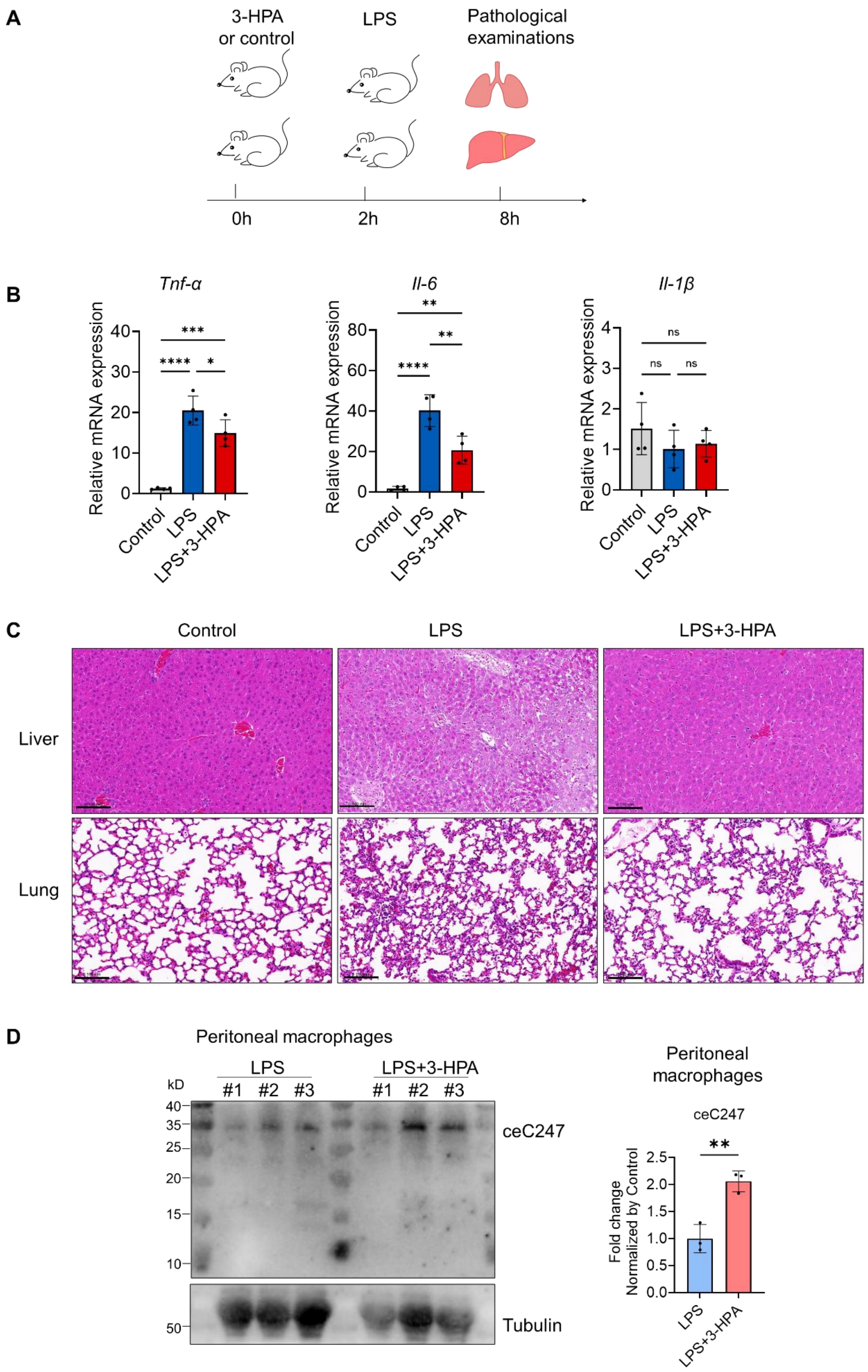

Fig S2. 3-HPA alleviates sepsis in LPS induced-model and promotes carboxyethylation modification of GAPDH in mouse peritoneal macrophages

(A) Schematic timeline of the experimental procedure. Mice were pretreated with 3-HPA or control, followed by LPS stimulation (2 h), and pathological examinations of lung and liver tissues were performed at 8 h post-LPS administration.

(B) qRT-PCR analysis of *Tnf-α, Il-6*, and *Il-1β* mRNA expression in the liver of LPS model (5 mg/kg) or LPS+3-HPA (50 mg/kg) (n=4 per group). Data are the means ± SD and n=4 per group. Statistical significance was determined using one-way ANOVA followed by Tukey's multiple comparisons test with $^{*}P<0.05$; $^{**}P<0.01$; $^{***}P<0.001$. $^{****}P<0.0001$; ns, not significant.

(C) HE staining of liver and lung tissues from mice treated with PBS, LPS, or LPS+3-HPA (scale bar: 100 μm).

(D) Immunoblotting showing ceC247 protein levels in LPS-stimulated macrophages treated with or without 3-HPA (n=3 biological replicates per group). Tubulin was used as the loading control. Right: Quantification of ceC247 protein expression, normalized to the LPS group and presented as fold change. Data are shown as mean ± SD and n=3 per group. Statistical significance was determined by Student's t-test: $^{**}P < 0.01$.

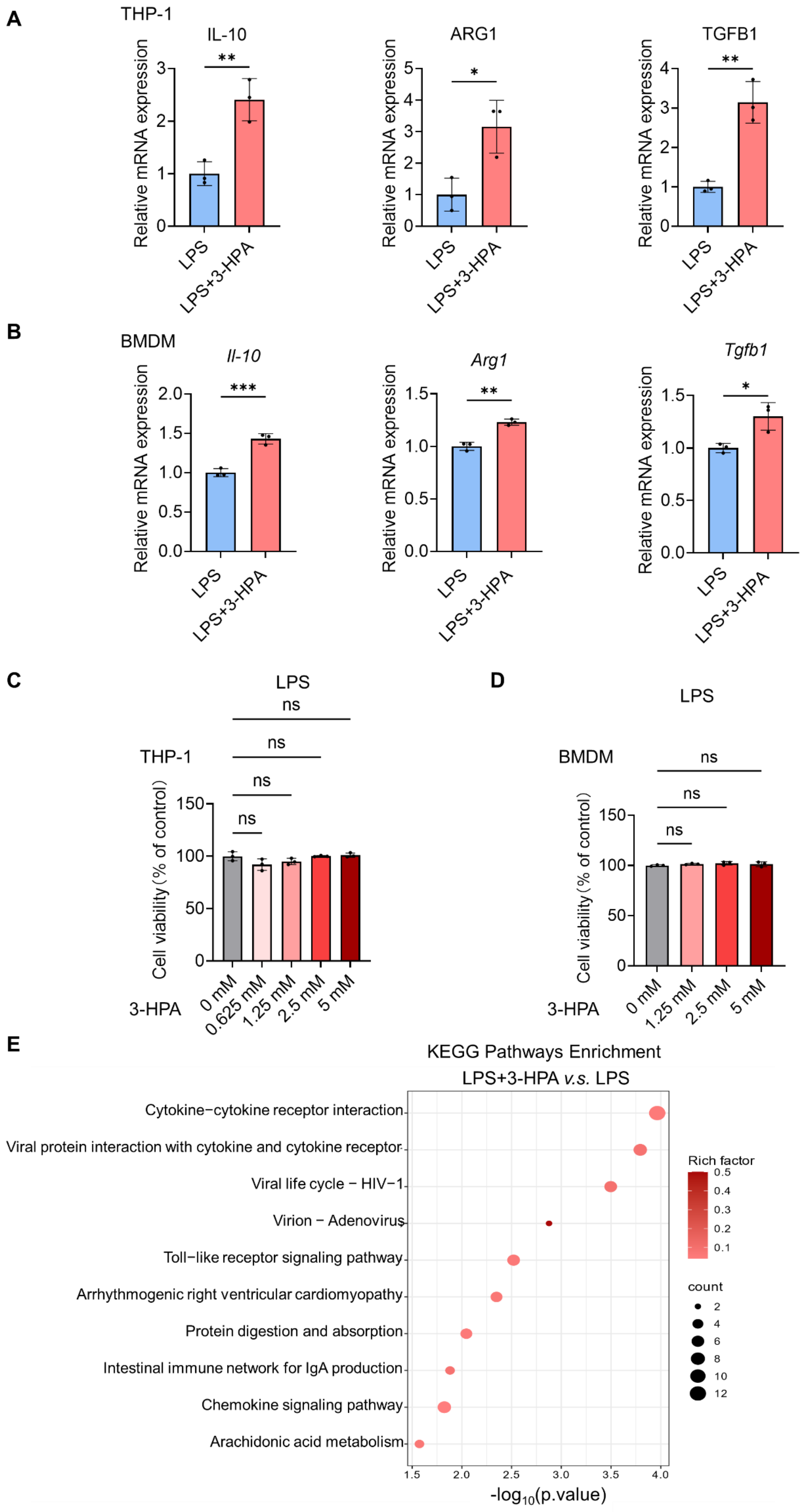
A
THP-1
IL-10
ARG1
TGFB1
Relative mRNA expression
LPS
LPS+3-HPA
B
BMDM
Il-10
Arg1
Tgfb1
C
LPS
THP-1
ns
Cell viability (% of control)
3-HPA
0 mM
0.625 mM
1.25 mM
2.5 mM
5 mM
D
BMDM
E
KEGG Pathways Enrichment
LPS+3-HPA v.s. LPS
Cytokine−cytokine receptor interaction
Viral protein interaction with cytokine and cytokine receptor
Viral life cycle − HIV−1
Virion − Adenovirus
Toll−like receptor signaling pathway
Arrhythmogenic right ventricular cardiomyopathy
Protein digestion and absorption
Intestinal immune network for IgA production
Chemokine signaling pathway
Arachidonic acid metabolism
Rich factor
count
-log10(p.value)

Fig S3. 3-HPA promotes the expression of anti-inflammatory related genes without affecting cell viability

(A) Relative mRNA expression of anti-inflammatory genes (*IL-10*, *ARG1*, *TGFB1*) in THP-1 macrophages treated with PBS or LPS, with or without 3-HPA supplementation. Data are presented as mean ± SD and n=3 per group. Statistical significance was determined by Student's t-test, *$P < 0.05$, **$P < 0.01$.

(B) Relative mRNA expression of anti-inflammatory genes (*Il-10, Arg1, Tgfb1*) in bone marrow-derived macrophages (BMDMs) treated with PBS or LPS, with or without 3-HPA supplementation. Data are presented as mean ± SD and n=3 per group. Statistical significance was determined by Student's t-test, *$P < 0.05$, **$P < 0.01$, ***$P < 0.001$.

(C) Cell viability of THP-1 macrophages treated with indicated concentrations of 3-HPA (0, 0.625, 1.25, 2.5, and 5 mM) in the presence of LPS (100 ng/mL), measured by CCK-8 assay and expressed as percentage of the 0 mM 3-HPA control group. Data are presented as mean ± SD and n=3 per group. Statistical significance was determined by one-way ANOVA followed by Dunnett's multiple comparisons test, ns, not significant.

(D) Cell viability of bone marrow-derived macrophages (BMDMs) treated with the same concentrations of 3-HPA under LPS stimulation. Data are presented as mean ± SD and n=3 per group. Statistical significance was determined by one-way ANOVA followed by Dunnett's multiple comparisons test, ns, not significant.

(E) Bubble plot of KEGG pathway enrichment analysis for differentially downregulated genes between LPS (100 ng/mL) and LPS+3-HPA (5 mM) treated in THP-1 cells. The size of each bubble represents the number of differentially expressed genes, and the color indicates the rich factor.

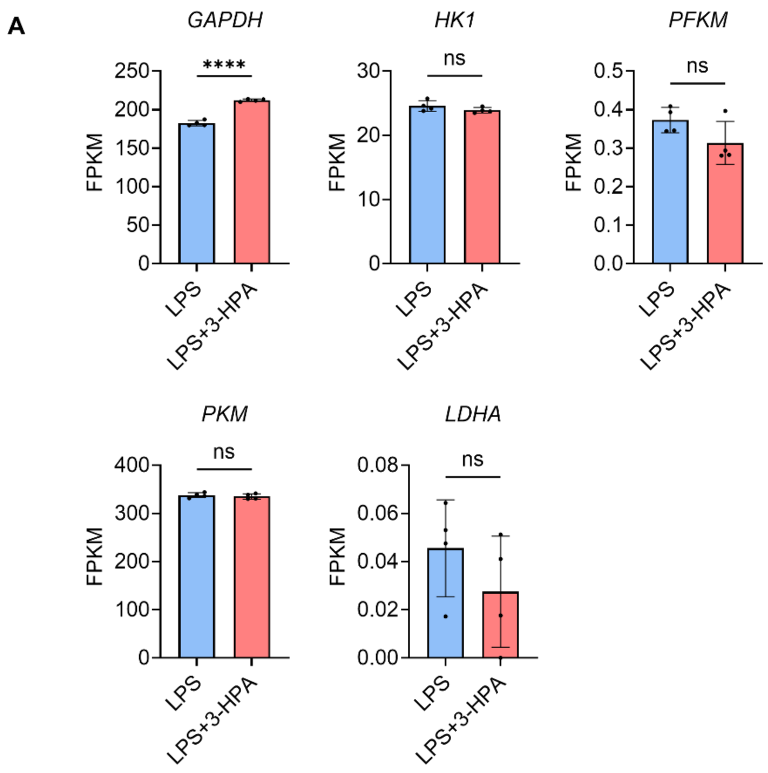

Fig S4. Effects of 3-HPA treatment on the expression of glycolytic genes

(A) RNA-seq analysis of FPKM expression for core glycolytic enzymes (*GAPDH, HK1, PFKM, PKM, LDHA*) in macrophages treated with LPS or LPS+3-HPA. Data are the means ± SD and n=4 per group. ****P < 0.0001; ns, not significant (Student's t-test).

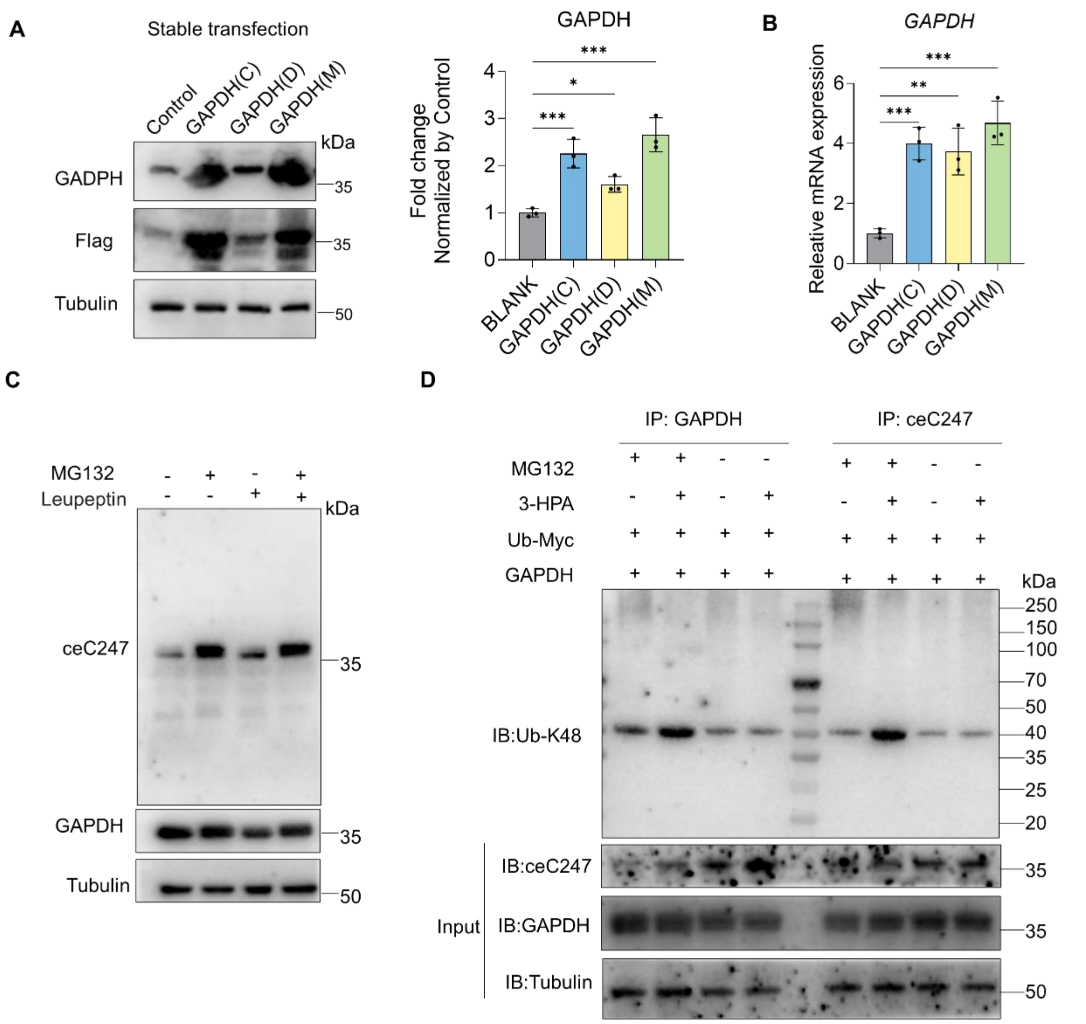

Fig S5. The carboxyethylation modification of GAPDH affects the expression of GAPDH

(A) Immunoblot and quantitative analysis of GAPDH after stable transfection of flag-tagged GAPDH(C), GAPDH(D), and GAPDH(M) in 293T cells. Data are the means ± SD and n=3 per group. Statistical significance was determined using one-way ANOVA followed by Dunnett's multiple comparisons test *P<0.05; **P<0.01; ***P<0.001.

(B) Relative mRNA expression of *GAPDH* was detected after stable transfection of flag-tagged GAPDH(C), GAPDH(D), and GAPDH(M) in 293T cells. Data are the means ± SD. Statistical significance was determined using one-way ANOVA followed by Dunnett's multiple comparisons test *P<0.05; **P<0.01; ***P<0.001.

(C) Immunoblot of GAPDH carboxyethylation (GAPDH ce) after treatment with lysosomal inhibitor leupeptin and MG132.

(D) Immunoprecipitation of GAPDH or GAPDH ce after treatment with MG132 and 3-HPA, followed by immunoblot for Ub-K48 in 293T cells transfected with Ub-Myc.

A

GAPDH Cys247 mutant to Glu247

Database	ΔΔG (kcal/mol)	Stability
mCSM	-1.059	Destabilizing
SDM	-1.18	Destabilizing
DUET	-0.917	Destabilizing

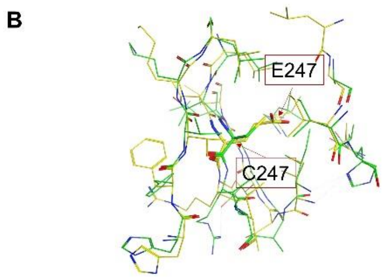

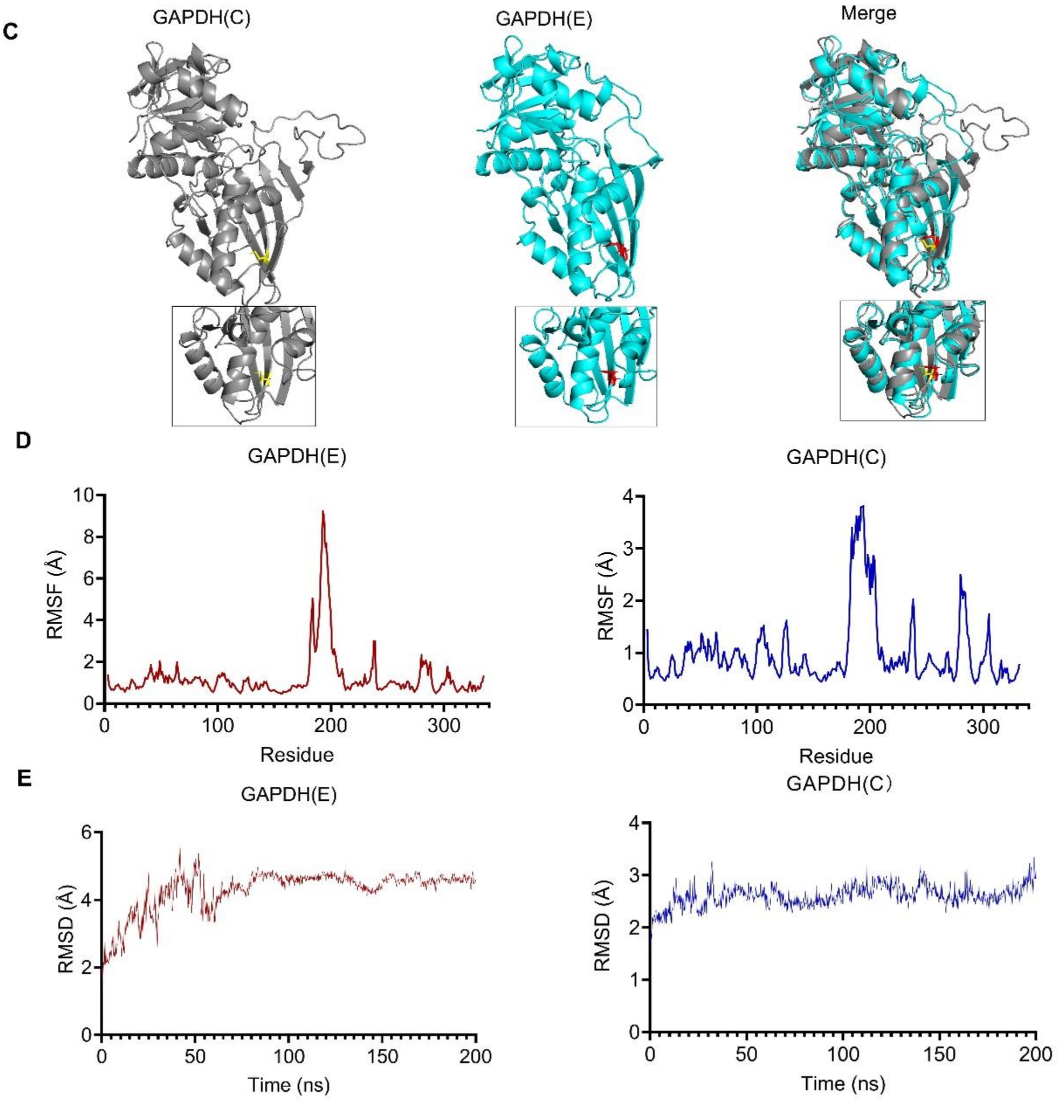

Fig S6. GAPDH carboxyethylation affects protein structure and stability

(A) Prediction of GAPDH stability change upon C→E mutation in the mCSM, SDM, DUET database.

(B) Structural comparison of GAPDH(C) and GAPDH(E) mutations at position 247.

(C) Ribbon diagrams of GAPDH(C) and GAPDH(E) protein structures, with merged view illustrating conformational differences between the wild-type and mutant forms.

(D) Molecular dynamics simulation calculations for root mean square fluctuation (RMSF) of GAPDH(E) and GAPDH(C).

(E) Molecular dynamics simulation calculations for root mean square deviation (RMSD) of GAPDH(E) and GAPDH(C).

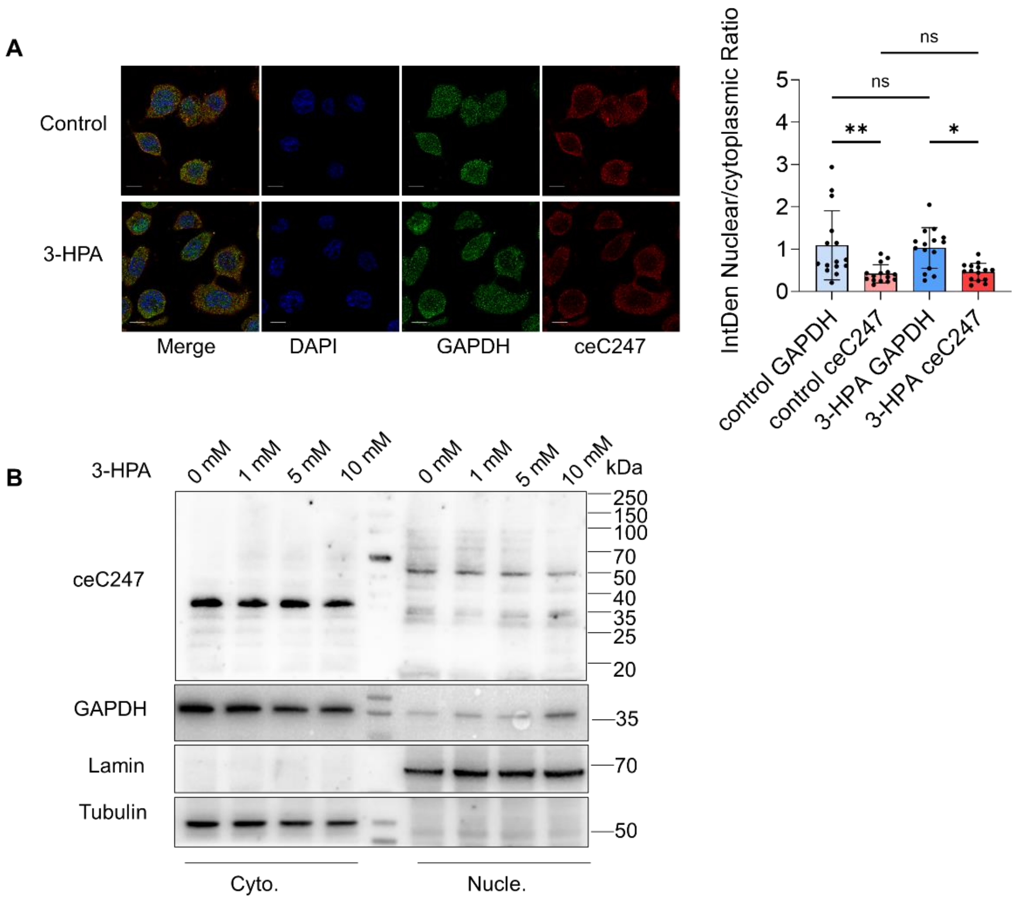

Fig S7. The GAPDH-ce molecule is located in the cytoplasm

(A) Immunofluorescence staining of GAPDH, GAPDH ce, and DAPI (nucleus) in THP-1 cells with or without 3-HPA treatment, along with quantitative analysis of nuclear/cytoplasmic ratio. Scale bars: 10 μm. Data are the means ± SD and n=15 per group. Statistical significance was determined using one-way ANOVA followed by Tukey's multiple comparisons test with $^{**}P<0.01$; ns, not significant.

(B) Immunoblot showing the nuclear and cytoplasmic localization, as well as the expression levels, of carboxyethylated GAPDH and total GAPDH in the presence of the 3-HPA.

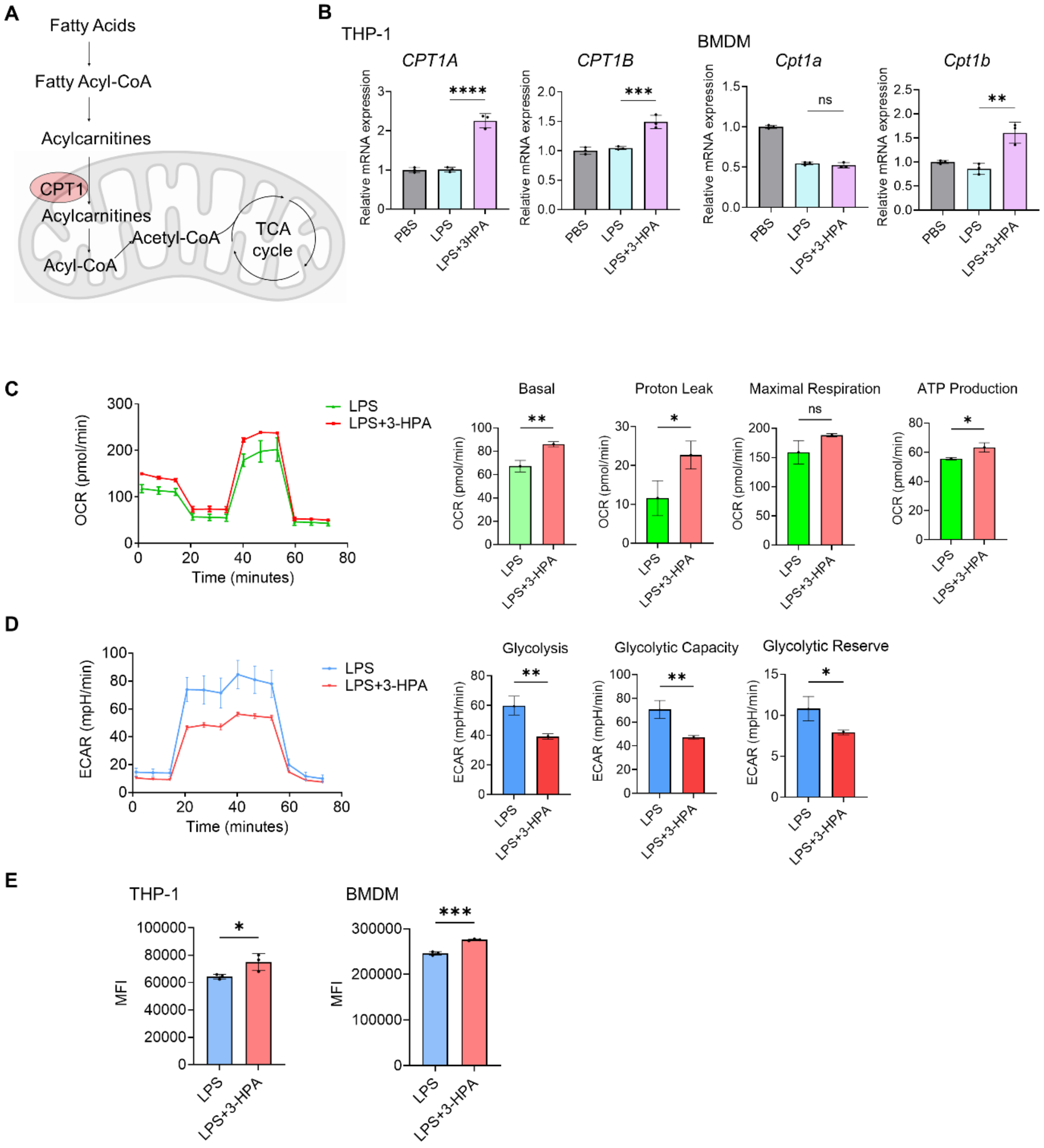

Figure S8. 3-HPA regulates macrophage energy metabolism and fatty acid oxidation

(A) Schematic illustration metabolic network of fatty acid β-oxidation and the TCA cycle.

(B) Relative mRNA expression of fatty acid oxidation-related genes *CPT1A* and *CPT1B* in THP-1 cells and BMDMs treated with PBS, LPS (100ng/ml), or LPS+3-HPA (5mM). Data are the means ± SD and n=3 per group. Statistical significance was determined using one-way ANOVA followed by Tukey's multiple comparisons test with *P<0.05; **P<0.01; ***P<0.001; ns, not significant.

(C) Oxygen consumption rate (OCR) was measured in LPS-activated BMDMs with or without 3-HPA treatment, and quantified for basal respiration, proton leak, maximal respiration, and ATP production. Data are shown as mean ± SD and n=3 per group. Statistical significance was determined by Student's t-test: *P < 0.05, **P < 0.01, ns, not significant.

(D) Extracellular acidification rate (ECAR) was assessed in BMDMs, and quantified for glycolysis, glycolytic capacity, and glycolytic reserve. Data are shown as mean ± SD and n=3

per group. Statistical significance was determined by Student's t-test: $*P < 0.05$, $**P < 0.01$.
(E) Mitochondrial mass was determined by TMRM and flow cytometry in THP-1(left) and BMDMs (right), presented as mean fluorescence intensity (MFI). Data are shown as mean ± SD and n=3 per group. Statistical significance was determined by Student's t-test: $*P < 0.05$, $***P < 0.001$; ns, not significant.

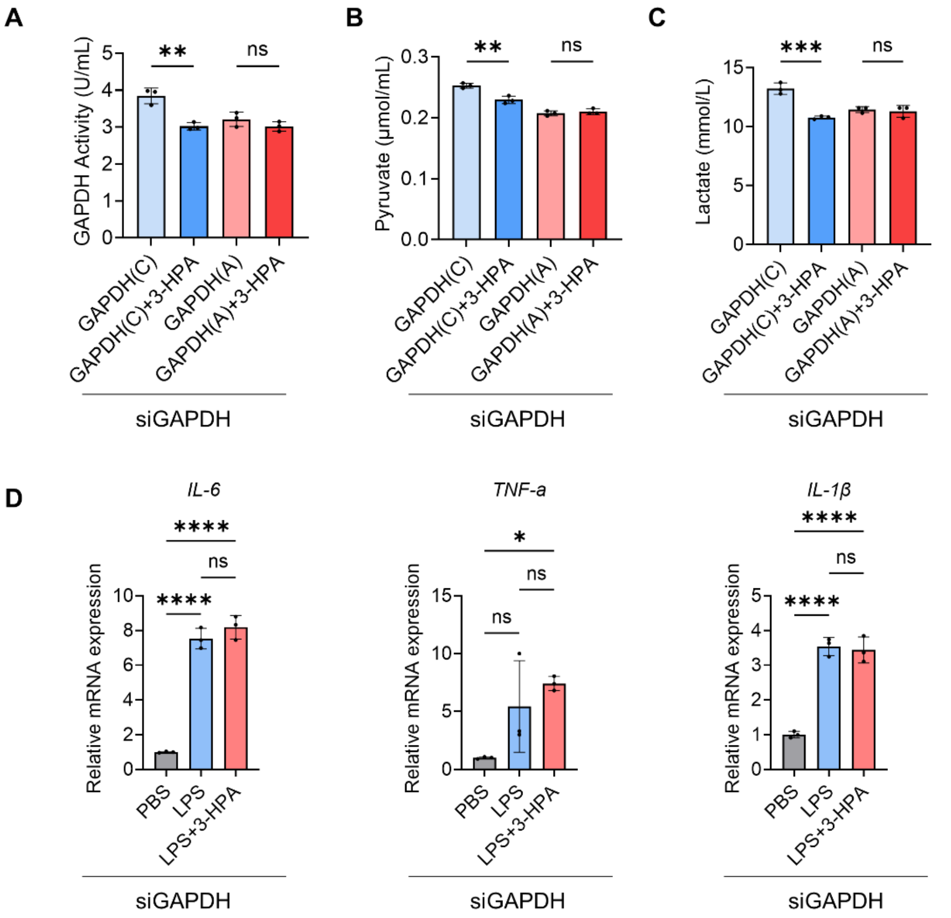

Figure S9. 3-HPA exerts its GAPDH enzyme activity and inflammation-suppressing functions dependent on GAPDH carboxyethylation at residue 247

(A-C) In GAPDH-knockdown THP-1 macrophages, (A) GAPDH enzymatic activity, (B) pyruvate production, and (C) lactate secretion were measured under conditions of overexpressing GAPDH(A) or GAPDH(C). Data are shown as mean ± SD and n=3 per group. Statistical analysis was performed using ANOVA with Tukey's multiple comparisons test: ***P*<0.01, ****P*<0.001, ns, not significant.

(D) In GAPDH-knockdown THP-1 macrophages, mRNA expression of pro-inflammatory cytokines (*IL-6, TNF-α, IL-1β*) was determined by qPCR under conditions of overexpressing GAPDH(A). Data are shown as mean ± SD and n=3 per group. Statistical analysis was performed using ANOVA with Tukey's multiple comparisons test: **P* < 0.05, *****P* < 0.0001; ns, not significant.
